# Tissue‐resident memory T cells in diseases and therapeutic strategies

**DOI:** 10.1002/mco2.70053

**Published:** 2025-01-12

**Authors:** Daoyuan Xie, Guanting Lu, Gang Mai, Qiuyan Guo, Guofeng Xu

**Affiliations:** ^1^ Laboratory of Translational Medicine Research Deyang People's Hospital of Chengdu University of Traditional Chinese Medicine Deyang China; ^2^ State Key Laboratory for Quality Ensurance and Sustainable Use of Dao‐di Herbs, Artemisinin Research Center, Institute of Chinese Materia Medica Academy of Chinese Medical Sciences Beijing China; ^3^ Inflammation & Allergic Diseases Research Unit The Affiliated Hospital of Southwest Medical University Luzhou China

**Keywords:** tissue‐resident memory T (T_RM_) cells, therapeutic targeting, neoadjuvant therapy, vaccine, immunotherapy

## Abstract

Tissue‐resident memory T (T_RM_) cells are crucial components of the immune system that provide rapid, localized responses to recurrent pathogens at mucosal and epithelial barriers. Unlike circulating memory T cells, T_RM_ cells are located within peripheral tissues, and they play vital roles in antiviral, antibacterial, and antitumor immunity. Their unique retention and activation mechanisms, including interactions with local epithelial cells and the expression of adhesion molecules, enable their persistence and immediate functionality in diverse tissues. Recent advances have revealed their important roles in chronic inflammation, autoimmunity, and cancer, illuminating both their protective and their pathogenic potential. This review synthesizes current knowledge on T_RM_ cells’ molecular signatures, maintenance pathways, and functional dynamics across different tissues. We also explore the interactions of T_RM_ cells with other immune cells, such as B cells, macrophages, and dendritic cells, highlighting the complex network that underpins the efficacy of T_RM_ cells in immune surveillance and response. Understanding the nuanced regulation of T_RM_ cells is essential for developing targeted therapeutic strategies, including vaccines and immunotherapies, to enhance their protective roles while mitigating adverse effects. Insights into T_RM_ cells’ biology hold promise for innovative treatments for infectious diseases, cancer, and autoimmune conditions.

## Graphical abstract

Tissue‐resident memory T (T_RM_) cells are crucial components of the immune system and play vital roles in antiviral, antibacterial, and antitumor immunity, as well as chronic inflammation conditions and graft‐versus‐host diseases. Understanding the intricate regulation of T_RM_ cells is crucial for developing targeted therapeutic strategies, such as vaccines and immunotherapies, that enhance their protective functions while minimizing potential adverse effects.

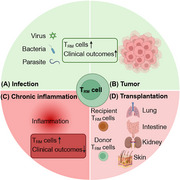

## 1 INTRODUCTION

The immune system's capacity to remember and respond more effectively to previously encountered pathogens is a cornerstone of adaptive immunity. This immunological memory is facilitated mainly by T cells, which differentiate into specialized subsets that provide durable protection. Among these subsets, tissue‐resident memory T (T_RM_) cells are crucial for immune surveillance, performing rapid and robust responses to reinfection at peripheral sites.^1^, ^2^

The subsets of T cells include circulating memory T cells, which are further divided into central memory T (T_CM_) cells, effector memory T (T_EM_) cells, and T_RM_ cells.^3^ Circulating memory T cells continuously survey the blood and lymphatic system for the presence of pathogens, remaining ready to initiate a rapid secondary immune response.^4^ In contrast, T_RM_ cells are a specialized subset that resides within tissues, offering immediate protection at sites of potential pathogen entry. T_RM_ cells have been identified in practically all tissues, including barrier sites (such as the skin, the intestine, the respiratory tract, and the urogenital tract),^5^, ^6^, ^7^, ^8^ primary and secondary lymphoid organs,^9^ and even nonbarrier tissues such as the brain and liver.^10^, ^11^ This widespread presence highlights their strategic role in immune surveillance and potential impact on disease outcomes. The strategic positioning and rapid response capabilities of T_RM_ cells render them promising for therapeutic applications. They offer the potential for enhancing vaccine efficacy through local immunity and for developing immunotherapies to target pathogens or cancer cells more effectively.^12^, ^13^

However, the use of T_RM_ cells for therapy presents challenges. Understanding the complex interplay between T_RM_ cells and tissue microenvironments, which influences their stability and functionality, is crucial.^14^, ^15^ Additionally, the heterogeneity of T_RM_ cells across different tissues and disease states complicates the development of one‐size‐fits‐all strategies.^16^, ^17^ Additionally, T_RM_ cells might contribute to chronic inflammation and autoimmunity when their activation is dysregulated.^18^ Advances in single‐cell sequencing and imaging technologies are revealing the molecular profiles and spatial distributions of T_RM_ cells in both health and disease.^19^, ^20^ These insights, along with a deeper understanding of T_RM_ cells’ maintenance, differentiation, and recall responses, pave the way for more targeted therapeutic strategies.^21^, ^22^ Given their abundance and adaptability, T_RM_ cells are key players in infection defense and the modulation of tissue‐specific immune responses. These roles make them crucial targets for strategies to enhance immune protection or treat diseases.

This review explores the roles of T_RM_ cells in various diseases, their mechanisms of action, and their therapeutic implications. We discuss the latest findings regarding T_RM_ cells’ behavior in different pathological contexts, the molecular pathways that govern T_RM_ cells’ development and maintenance, and the current landscape of therapeutic approaches targeting T_RM_ cells. By integrating insights from diverse fields, we aim to provide a comprehensive overview of T_RM_ cells as guardians and mediators of tissue immunity and as emerging targets for the treatment of a spectrum of diseases.

## 2 BIOLOGICAL CHARACTERISTICS OF T_RM_ CELLS

Upon encountering a pathogen, naïve CD8^+^ T cells transform into effector cells that generate inflammatory cytokines and cytotoxic granules for pathogen elimination. After infection, most antigen‐specific T cells are eliminated, but some persist as memory T cells within tissues, forming two groups, circulating memory T cells in the bloodstream and lymphoid tissues and T_RM_ cells in peripheral tissues, to offer long‐lasting, localized immunity.^23^, ^24^, ^25^ The formation and maintenance of T_RM_ cells are critical for long‐term immunological memory and are characterized by a distinct set of biomarkers, as detailed in the following sections.

### 2.1 Origin and formation of T_RM_ cells

T_RM_ cells originate from the activation of naïve T cells, which, upon encountering antigens, differentiate into various subsets, including effector and memory T cells.^26^ The differentiation into T_RM_ cells is a critical process that involves a series of cell fate decisions governed by a complex interplay of transcriptional regulators and environmental cues. Memory T cells further diversify into T_EM_ cells, T_CM_ cells, and T_RM_ cells, which are distinguished by their residence in peripheral tissues. Recent epigenetic studies have shown that long‐lived memory CD8^+^ T cells originate from a subset of effector CD8^+^ T cells that are capable of re‐expressing genes typically associated with a naïve state, facilitated by an open chromatin state that allows rapid effector function upon re‐encountering an antigen.^21^, ^27^

The formation of T_RM_ cells is intimately linked to the expression of CD127 and killer cell lectin‐like receptor G1 (KLRG1) on effector T cells.^28^ CD127^high^ effector T cells, identified as memory precursor effector cells (MPECs), are particularly adept at giving rise to both resident and circulating memory T cells.^29^, ^30^ These cells express high levels of antiapoptotic molecules, enabling them to persist and provide long‐term protective immunity. In contrast, KLRG1^high^CD127^low^ cells give rise to short‐lived effector cells (SLECs), which are important for immediate immune responses but do not contribute to long‐term immunity.^30^ Longitudinal tracking of T cells has revealed the marked developmental plasticity of KLRG1^high^CD8^+^ effector T cells. These cells have the capacity to downregulate KLRG1 expression in a Bach2‐dependent manner, allowing them to differentiate efficiently into a variety of memory T‐cell lineages.^31^ This adaptive response endows them with versatility that makes them highly effective in both antiviral and antitumor immunity. A pivotal role in T_RM_ cells’ formation is played by the transcription factors Hobit and Blimp1, whose expression suppresses the expression of genes related to tissue egress, thereby promoting a transcriptional program conducive to tissue residency.^32^ Additionally, microenvironmental cues, such as transforming growth factor β (TGF‐β) and interleukin 15 (IL‐15) expression, are essential for the formation of long‐lived memory T cells and their specific localization within tissues.^33^, ^34^

In essence, the emergence of T_RM_ cells is a tightly controlled process shaped by intrinsic cellular programs and extrinsic environmental factors, ultimately producing a subset of T cells uniquely adapted for tissue‐specific immunity.

### 2.2 Cellular biomarkers of T_RM_ cells

T_RM_ cells exhibit unique surface markers that are critical for their tissue‐specific homing and retention. Key markers include CD103, CD69, CD49a, and CXCR6 expression, which enable T_RM_ cells to inhabit peripheral tissues such as the intestine, skin, and liver, where they perform immunosurveillance and exhibit enhanced antigen recall capacity. Although T_RM_ cells possess a set of fundamental transcriptional markers, tissue‐specific microenvironments induce organ‐specific genetic responses, allowing T_RM_ cells to adapt to their tissues of residence.

#### 2.2.1 CD103

CD103, also known as αEβ7 integrin, is a crucial marker for T_RM_ cells that assists in their retention, cytotoxicity, and interactions within the tumor microenvironment (TME). Regulated by the expression of the transcription factors RUNX2 and RUNX3, CD103 binds to E‐cadherin on epithelial cells, facilitating T_RM_ cells’ localization in epithelial tumor regions.^35^, ^36^, ^37^ CD103 expression varies across tissues, and its upregulation in mucosal tissues such as the reproductive tract, skin, lungs, small intestine, and salivary glands is essential for its retention and function.^38^, ^39^, ^40^ In contrast, T_RM_ cells in tissues such as the lamina propria of the small intestine or internal organs such as the kidneys or liver often lack CD103 expression and rely instead on the expression of alternative retention molecules such as VLA‐1 (integrin α1β1) and LFA‐1 (integrin αLβ2).^8^

#### 2.2.2 CD69

The expression of CD69, a C‐type lectin, is important for the retention of T_RM_ cells in peripheral tissues. Although CD69 is commonly employed as a marker for identifying T_RM_ cells during homeostatic conditions, its expression is subject to significant fluctuations and can be triggered by numerous stimuli.^41^, ^42^ By acting as an antagonist of sphingosine‐1‐phosphate receptor 1 (S1P1), CD69 expression curbs the egress of T cells by impeding their responsiveness to sphingosine‐1‐phosphate (S1P) gradients, thereby promoting T‐cell residence in tissues.^43^, ^44^ Despite its importance, CD69 expression is not essential for the existence and retention of T_RM_ cells, indicating that multiple retention factors maintain T_RM_ cells’ populations. Its primary function may be to restrict the exit of T cells from organs, but its precise role in tissue‐specific immunity requires further investigation.

#### 2.2.3 CD49a

CD49a, the α1‐subunit of integrin α1β1, binds to collagen I/IV, enhancing T‐cell attachment to the extracellular matrix and initiating cytotoxicity and interferon‐γ (IFN‐γ) production.^45^, ^46^ CD49a is expressed by T_RM_ cells in tissues such as the lungs, skin, and liver.^47^, ^48^, ^49^ It is regulated by the expression of RUNX2 and RUNX3, which promote the expression of genes that are essential for immune surveillance.^48^, ^50^, ^51^ CD49a^+^ T_RM_ cells often coexpress CD103, which aids in epithelial retention by binding to E‐cadherin.^52^ They also show limited TCR repertoire diversity, which is indicative of clonal expansion and tissue‐specific differentiation.^53^ Advances in single‐cell RNA sequencing and ATAC sequencing have detailed the molecular and epigenetic profiles of CD49a^+^ T_RM_ cells, highlighting their metabolic reprogramming toward mitochondrial β‐oxidation.^21^

#### 2.2.4 CXCR6

CXCR6, a chemokine receptor that binds to CXCL16, has emerged as a distinctive molecular biomarker for T_RM_ cells and is essential for immunosurveillance. CXCR6 interacts with dendritic cells (DCs) within tumors, promoting T_RM_ cells’ proliferation and enhancing antitumor vaccination efficacy. CXCR6 and CD38 expression remain stable under inflammatory conditions, making these molecules reliable markers for T_RM_ cell characterization.^19^ CXCR6 is crucial for generating T_RM_ cells, particularly TGF‐β‐dependent CD103^+^ T_RM_ cells.

To identify additional markers for T_RM_ cells, Mackay and colleagues performed uniform manifold approximation and projection (UMAP) analysis and reported that, in addition to CD69, liver T_RM_ cells also express CD38, CD39, CD85k, and CXCR6. Further analysis revealed that T_RM_ cells in the kidneys also express CD69, CD38, CD39, and CXCR6.^19^, ^54^ Despite significant disparities in transcriptional profiles and migration patterns, the precise characterization and targeted manipulation of T_RM_ cells remain a formidable challenge. In particular, identifying reliable markers for T_RM_ cells remains challenging, which emphasizes the need for the further development of such tools to advance the understanding of T_RM_ cell biology.

While investigating the surface markers of T_RM_ cells, we noted that, in addition to the expression of key molecules such as CD103 and CD69, the expression of several other molecules significantly contributes to the functionality and tissue‐specific localization of T_RM_ cells. To provide a comprehensive understanding of the molecular characteristics of T_RM_ cells, we have categorized and described these molecules in the table below (Table 1).

**TABLE 1:** T_RM_ cells’ phenotypic markers.

Feature category	Molecule name	Functional description
Transcription factors	BLIMP1^32^	Collaborates with the related transcription factor Hobit to synergistically promote T_RM_ cell development, maintain tissue residency, and repress genes required for tissue egress.
	HOBIT^32^	This transcription factor is upregulated in T_RM_ cells and is essential for T_RM_ cell development in the skin, gut, liver, and kidney, cooperating with Blimp1 to mediate tissue residency.
	RUNX3^55^	Runx3 promotes CD8^+^ T_RM_ cell development and viability; increasing its levels may enhance treatment strategies for infectious diseases and cancer.
Tissue adhesion and retention molecules	CD103^36^	Also known as the αE‐subunit of integrin αEβ7, CD103 interacts with E‐cadherin on epithelial cells, promoting T‐cell binding and retention in tissues.
	CD69^43^	Acts as an antagonist to S1P1, reducing T‐cell egress by limiting responsiveness to S1P gradients, thus promoting tissue residency. Downregulation of S1P1 and KLF2 in T_RM_ cells, combined with sustained expression of CD69, promotes retention rather than circulation.
	CD49a^56^	As part of α1β1 integrin, CD49a binds to collagen, increasing T‐cell adhesion to the extracellular matrix, promoting tissue retention, and preparing for cytotoxic activity and IFN‐γ production.
	CD44^57^	CD44 is a C‐lectin‐containing glycoprotein that serves as a receptor for hyaluronic acid. It plays a role in cell structure maintenance and migration through adherence to extracellular matrix proteins and potentially aids in the retention and positioning of T_RM_ cells within peripheral tissues.
	CD101^58^	CD101 is selectively expressed on CD8^+^ T_RM_ cells across various sites, suggesting a role in tissue‐specific homing and retention, and could serve as a marker for T_RM_ cells and have immunomodulatory functions.
Chemokine receptors	CCR9^59^	CCR9 is found on some T cells, including T_RM_ cells, and interacts with its ligand CCL25 in the small intestinal epithelium. It aids in inducing CD103 on CD8^+^ intraepithelial T lymphocytes and is crucial for T_RM_ cell localization and function in the small intestinal mucosa.
	CXCR6^19^	A chemokine receptor that binds CXCL16 and a marker identifying a T_RM_ cell subset involved in immunosurveillance. It is crucial for interactions with tumor dendritic cells, promoting the proliferation of CXCR6^+^ T_RM_ cells and enhancing the efficacy of antitumor vaccines and chimeric antigen receptor‐T‐cell therapies.
	CX3CR1^58^	CX3CR1, a chemokine receptor, is downregulated in human CD69^+^ T_RM_ cells, indicating its potential role in reducing the migratory capacity of T_RM_ cells toward its ligand CX3CL1, thus promoting their tissue residency.
Lymphoid homing molecules	CD62L^58^	CD62L, also known as L‐selectin, is downregulated in human CD69^+^ T_RM_ cells, suggesting a role in limiting the recirculation of these cells to lymph nodes and promoting their retention in peripheral tissues.
	CCR7^60^	CCR7 expression is typically low or absent in T_RM_ cells, suggesting a role in limiting the recirculation of T_RM_ to lymphoid tissues and facilitating their retention in peripheral tissues.
	S1PR1^61^	S1P1 binds S1P to guide T_RM_ cell migration along its gradient, but CD69 antagonizes S1P1, reducing migration and promoting T_RM_ cell retention in tissues.

### 2.3 Maintenance and activation of T_RM_ cells

The persistence of T_RM_ cells in peripheral tissues is fundamental to their role in long‐term immunological memory and protection against recurrent infections. T_RM_ cells persist in peripheral tissues by adapting to local cues and resisting dispersal signals.^62^ One key mechanism involves interactions with epithelial cells via the integrin αEβ7, which binds E‐cadherin, potentially anchoring T_RM_ cells and promoting their survival through the expression of prosurvival proteins such as Bcl‐2.^33^, ^40^, ^63^ However, the role of αEβ7 is context‐dependent, and this molecule may not be necessary for T_RM_ cells maintenance in all tissues. Another critical factor is the regulation of S1PR1, which mediates T‐cell egress from the lymph nodes.^61^ T_RM_ cells downregulate S1PR1 and upregulate CD69 expression to prevent egress and promote tissue residency. The expression of the transcription factor KLF2, which influences S1PR1 expression, is downregulated in T_RM_ precursors upon tissue entry. Additionally, other S1P receptors, such as S1PR5, may play tissue‐specific roles in T_RM_ cell behavior.

Memory T_RM_ cells maintain long‐term immune protection through selective homing and positioning facilitated by adhesion molecules and chemokine receptors. Their retention and metabolic adaptability are supported by the stable expression of markers such as CD69 and CD103. Epigenetic modifications, including DNA methylation and histone modifications, play crucial roles in maintaining T_RM_ cell‐specific gene expression profiles.^64^ Self‐renewal mechanisms and interactions with tissue‐resident cells provide survival signals and nutritional support, further increasing T_RM_ cells’ persistence.^65^

Upon re‐encounter with pathogens or, upon tumor recurrence, T_RM_ cells swiftly initiate an immune response within infected tissues. They maintain their tissue residency through the expression of specific transcription factors such as Hobit, which collaborates with Blimp‐1 to suppress pathways that facilitate tissue egress while promoting tissue retention.^22^ They recognize antigens, expand rapidly and locally, and produce proinflammatory cytokines such as IFN‐γ and TNF‐α to eradicate pathogens. Some T_RM_ cells differentiate into circulating T_EM_ cells that patrol the body for new threats, whereas others migrate to draining lymph nodes to form secondary T_RM_ cells.^66^ These processes collectively position T_RM_ cells as crucial components of early immune defense, providing effective protection during recurrent infections.

Understanding the nuanced regulatory mechanisms of T_RM_ cell maintenance and activation is pivotal for developing strategies to enhance their protective functions while minimizing potential adverse effects in chronic inflammatory conditions. This knowledge has important implications for vaccine development and targeted immunotherapies.

### 2.4 Differentiation pathways of T_RM_ cells

The differentiation of T_RM_ cells into tissue‐specific subsets integrates intrinsic cellular programs with environmental signals, shaping the roles of these cells in local immune responses against infections and tumors. This section summarizes key insights from recent studies on T_RM_ cell differentiation.

The initial differentiation of T_RM_ cells is influenced by the priming of naïve T cells in lymph nodes by DCs, which present pathogen‐derived peptides, with cytokines such as TGFβ playing a pivotal role.^67^ Research by Carbone emphasized the role of RUNX3 as a key transcription factor that, in conjunction with TGFβ signaling, directs the development and maintenance of CD8^+^ T_RM_ cells, particularly in nonlymphoid tissues.^7^ RUNX3 expression orchestrates the expression of genes involved in tissue retention and immune function, and TGFβ expression is a critical upstream signal. This signaling axis is essential for suppressing the expression of tissue egress receptors such as S1PR1 and promoting the expression of tissue retention molecules such as CD103.^68^ T_RM_ cells diversify into subsets adapted to their tissue microenvironments. For example, in the urogenital tract, T_RM_ cells must adapt to a unique set of challenges, including sexually transmitted infections and tumor surveillance, which require specific differentiation influenced by local TGFβ and cytokine expression.^8^ In the lungs, the transient nature of T_RM_ cell residency may be a result of organ‐specific features that prioritize the delicate balance between immune protection and tissue integrity.^69^

Understanding the differentiation process of T_RM_ cells is crucial for leveraging their potential in immunotherapy and vaccine development. Future research should explore the nuances of T_RM_ cell differentiation to enhance their protective functions while minimizing adverse effects in chronic inflammatory conditions.

## 3 DISEASE‐FIGHTING MECHANISMS OF T_RM_ CELLS

### 3.1 Immune surveillance

T_RM_ cells play a crucial role in immune surveillance by maintaining a constant presence in tissues to quickly detect and respond to infections and tumor development. T_RM_ cells can secrete effector molecules such as IFN‐γ, TNF, and IL‐2, which not only activate local immune responses but also recruit additional immune cells to bolster defense.^70^ Because they are equipped with cytotoxic molecules such as perforin and granzyme, T_RM_ cells possess the unique ability to directly eliminate tumor cells. By binding to E‐cadherin on tumor cells through CD103 expression, T_RM_ cells stabilize their immunological synapses and consequently increase their cytotoxic efficiency.^71^ Furthermore, T_RM_ cells contribute to maintaining tumor–immune equilibrium, thereby curbing tumor growth and spread. Their memory function provides long‐term protection, making them vital for counteracting recurring threats. The unique metabolic features and rapid response capabilities of T_RM_ cells underscore their essential role in adaptive immunity as a first line of defense against various pathogens and tumors. Delving into the dynamics of T_RM_ cells activity reveals that their role clearly transcends mere surveillance. In the elimination phase, T_RM_ cells actively engage and destroy tumor cells, either directly through cytotoxic mechanisms or indirectly by modulating the immune microenvironment. Despite potential exhaustion, they persist in the equilibrium phase, maintaining a balance that hinders tumor advancement. However, if T_RM_ cells succumb to senescence, the escape phase becomes a reality as the tumor maneuvers to evade weakened immune control, which underscorse the imperative need to understand and bolster T_RM_ cells’ function in cancer immunotherapy strategies.^72^

### 3.2 Cellular interactions

#### 3.2.1 Interaction between T_RM_ cells and macrophages

**FIGURE 1:**
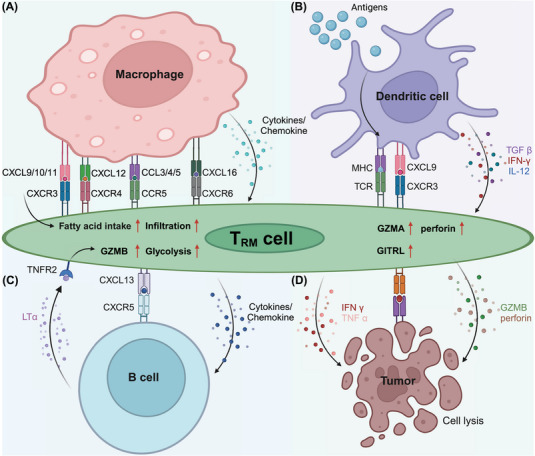
Cellular interactions of T_RM_ cells. (A) In the TME, macrophages actively participate in the recruitment of T_RM_ cells through the CXCL9/10/11‐CXCR3, CXCL12‐CXCR4, and CCL3/4/5‐CCR5 signaling pathways. They also provide T_RM_ cells with essential fatty acids to sustain them. Resident alveolar macrophages (Res‐AMs) express CXCL16, and the chemokines and cytokines they release promote the accumulation of CXCR6^+^ T_RM_ cells within the human lung TME. (B) DCs express high levels of MHC molecules (both MHC‐I and MHC‐II), which present tumor antigens to T_RM_ cells via peptide‐MHC complexes. DCs‐activated T_RM_ cells expressed core components of the cytotoxic machinery like GZMA and perforin 1(PRF1). DCs also stimulate the expression of the tissue‐homing molecule CD103 on CD8^+^ T cells and, following exposure to type I interferons, upregulate the co‐stimulatory molecule GITRL, which supports T_RM_ cell formation. By increasing chemokine expression, especially CXCL9, DCs boost the anti‐tumor activity of T_RM_ cells and recruit CXCR3^+^ T_RM_ cells. Additionally, DCs provide metabolic support to T_RM_ cells through the secretion of fatty acids and other metabolites. In inflammatory environments, DCs secrete cytokines such as IL‐12, IFN‐γ, and TGF‐β, promoting the infiltration, accumulation, and retention of T_RM_ cells in tissues and thereby enhancing localized immune responses. (C) Activated B cells enhance the expression of CXCL13 and the secretion of GZMB in T_RM_ cells through the lymphotoxin‐α/tumor necrosis factor receptor 2 (TNFR2) axis, and they also promote glycolysis in T_RM_ cells via this same pathway. (D) Activated T_RM_ cells secrete IFN‐γ and TNF‐α and express cytotoxic molecules such as GZMB and PFN, further promoting the lysis of tumor cells. This figure was designed using BioRender (https://biorender.com/).

T_RM_ cells and lung‐resident macrophages (MLRs) engage in interactions that are essential for immune surveillance and response in both homeostatic and disease contexts (Figure 1). Using ex vivo lung perfusion (EVLP) technology, researchers have discovered that human lung T_RM_ cells and MLRs colocalize, particularly around airways, where they participate in intricate immunological interactions. Mechanistically, MLRs interact with highly PD‐1‐expressing T_RM_ (PD‐1^high^ T_RM_) cells by providing essential costimulatory signals.^73^ This interaction is pivotal, as it promotes the expression of the activation marker CD107a on the surface of PD‐1^high^ T_RM_ cells, thereby priming these cells for a more effective immune response. Furthermore, this interplay between MLRs and PD‐1^high^ T_RM_ cells promotes the release of crucial cytokines, such as IFNγ and TNFα. These cytokines play pivotal roles in recruiting and activating other immune cells, reinforcing the local immune response.

In the non‐small‐cell lung cancer (NSCLC) TME, a subset of tumor‐associated macrophages (TAMs), characterized by an M1‐like phenotype (M1^hot^ TAMs), has been found to significantly increase T_RM_ cell infiltration and survival.^74^ These M1^hot^ TAMs express high levels of chemokines, including CXCL9, which are critical for attracting activated TH1 T cells and T_RM_ cells expressing the chemokine receptor CXCR3. Moreover, M1^hot^ TAMs provide the essential fatty acids for the sustenance of T_RM_ cells, facilitating their infiltration and function within the tumor. The presence of M1^hot^ TAMs is correlated with a greater density of CD8^+^ T_RM_ cells within the tumor and is associated with improved patient outcomes. However, the TME is highly heterogeneous, and the presence of M2‐like TAMs can present a significant challenge. M2‐like TAMs, which are typically associated with immunosuppression, compete with T_RM_ cells for critical nutrients such as fatty acids, potentially impairing the long‐term survival and antitumor efficacy of T_RM_ cells.^75^ This competitive dynamic underscores the importance of the TAM phenotype in shaping the overall immune response against cancer. Indeed, M2‐like TAMs contribute to a tumor‐promoting microenvironment by secreting anti‐inflammatory cytokines such as IL‐10 and TGF‐β, which can dampen the activity of T_RM_ cells and hinder their ability to control tumor progression. Furthermore, recent studies have suggested that the polarization of macrophages toward the M1 or M2 phenotype is not static but can be dynamically regulated by environmental cues within the TME. Therefore, therapeutic strategies that target the modulation of TAM polarization, particularly those that promote the M1 hot phenotype, could be pivotal in enhancing T_RM_ cell‐mediated antitumor immunity. For example, therapies designed to increase CXCL9 expression or fatty acid availability within the TME could improve T_RM_ cells’ function and survival, providing a new avenue for cancer immunotherapy.

In summary, the interaction between T_RM_ cells and macrophages is a key determinant of immune efficacy in both homeostasis and cancer. By understanding the molecular mechanisms that govern these interactions, we can develop targeted therapeutic strategies that promote the beneficial aspects of TRM–macrophage crosstalk while mitigating the suppressive influences of M2‐like TAMs. These findings highlight the potential of modulating the T_RM_–macrophage axis as a promising approach for improving immunotherapeutic outcomes in cancer.

#### 3.2.2 Interaction between T_RM_ cells and DCs

In the TME and other pathological conditions, the intricate interplay between T_RM_ cells and DCs is pivotal for the initiation and modulation of immune responses (Figure 1). As key antigen‐presenting cells, DCs not only play a role in the initial differentiation of T_RM_ cells but also contribute to their maturation and functional maintenance by producing essential costimulatory signals and cytokines, such as CD24, IL‐12, and IL‐15.^76^ These cytokines activate specific signaling pathways in T_RM_ cells, including STAT4 and STAT5, which are vital for their long‐term persistence and effector functions. Additionally, DCs regulate the metabolic reprogramming of T_RM_ cells, ensuring their ability to adapt to the nutrient‐deprived microenvironment of inflamed tissues or tumors.^77^ Particularly in secondary lymphoid organs, the cDC1 subset is crucial for the generation of T_RM_ cell precursors through cross‐presentation mechanisms.^78^ This cross‐presentation allows cDC1s to efficiently present tumor‐associated antigens to naïve CD8^+^ T cells, leading to the differentiation of T_RM_ cell precursors that later migrate to peripheral tissues. Once in tissues, T_RM_ cells interact with local DCs, which further modulate their phenotype and enhance their immune surveillance capabilities. For example, in inflammatory conditions, DCs secrete a range of cytokines, including IL‐12, IFN‐γ, and TGF‐β, that promote T_RM_ cell infiltration, accumulation, and retention within the tissue, thereby enhancing local immune responses.

Tumor‐associated DCs (TADCs) modulate the phenotypes and functions of T_RM_ cells, thereby influencing their proliferation, survival, and expression of effector molecules.^79^ TADCs with an M1‐like phenotype significantly increase the antitumor activity of T_RM_ cells by increasing the expression of chemokines such as CXCL9, which recruits T_RM_ cells that express CXCR3. These M1‐like TADCs also provide metabolic support to T_RM_ cells by secreting fatty acids and other metabolites that are crucial for their energy production and survival. This interaction fosters a positive feedback loop that amplifies local immune responses, leading to greater T_RM_ cell accumulation and enhanced antitumor effects. On the other hand, M2‐like TADCs, which are typically associated with immunosuppressive environments, counteract these effects by releasing cytokines such as IL‐10 and TGF‐β, which inhibit T_RM_ cell activation and proliferation. Moreover, M2‐like TADCs may compete with T_RM_ cells for nutrients, further limiting their effector functions and undermining their ability to control tumor growth. Modulating the activities and phenotypes of DCs can promote the effector functions of T_RM_ cells, improving the efficacy of immunotherapy. For example, vaccines or immunomodulators that activate DCs could promote the generation and maintenance of T_RM_ cells, thereby strengthening their immune surveillance and tumor elimination capabilities.^80^ These strategies might involve the use of specific molecules such as CXCL9 to attract T_RM_ cells or the modulation of cytokines released by DCs to augment T_RM_ cell activity. Targeting the T_RM_ cells‒DC axis could be key to enhancing the outcomes of immunotherapy.

The interaction between T_RM_ cells and DCs is not limited to the TME but extends to other pathological conditions, including chronic infections and autoimmune diseases. In these contexts, DCs can promote protective immune responses or contribute to tissue damage, depending on the inflammatory milieu and the specific DC subsets involved. Therefore, targeting the T_RM_ cell‒DC axis could be key not only for enhancing the outcomes of cancer immunotherapy but also for managing chronic inflammatory diseases and infections. Future research should focus on identifying specific DC subsets and their roles in regulating T_RM_ cells in different tissue contexts and on developing targeted therapies that modulate these interactions for therapeutic benefit.

#### 3.2.3 Interaction between T_RM_ cells and B cells

T_RM_ cells interact with other immune components in the TME, significantly influencing the effectiveness of immunotherapy (Figure 1). One of their key interactions is with B cells, particularly those found in tertiary lymphoid structures (TLSs). These structures often form within tumors and play a pivotal role in orchestrating local immune responses. For example, in gastric cancer, B cells within TLSs promote glycolysis in CD103^+^CD8^+^ T_RM_ cells through the lymphotoxin‐α (LTα)/tumor necrosis factor receptor 2 (TNFR2) signaling axis, increasing their metabolic adaptability and thereby boosting the effector functions of T_RM_ cells for an effective antitumor response.^81^ The ability of B cells to modulate T_RM_ cell activity through metabolic reprogramming underscores their importance in sustaining long‐term immune surveillance and promoting the persistence of T_RM_ cells in the TME. Additionally, although they are less well characterized than T_RM_ cells, tissue‐resident memory B (B_RM_) cells play a complementary role in immune defense. Studies have shown that B_RM_ cells, particularly in mucosal tissues, can respond to local antigen exposure and contribute to rapid antibody production. This interaction between B_RM_ and T_RM_ cells may create a synergistic effect in which B_RM_ cells provide early humoral responses, while T_RM_ cells sustain long‐term cellular immunity. In models of pulmonary infections, B_RM_ cells have been shown to accelerate immune responses by quickly differentiating into plasma cells upon antigen re‐encounter, producing cross‐neutralizing antibodies against diverse pathogens.^82^ This rapid response is particularly important in tissues where maintaining an immediate and robust immune response is critical for pathogen clearance.

In contrast, in HPV‐positive oropharyngeal squamous cell carcinoma (OPSCC), T_RM_ cells expressing high levels of CD161 exhibit an inhibitory effect on TLS activity.^83^ The upregulation of CD161 expression on T_RM_ cells correlates with a reduction in the expression of CLEC2D, the CD161 ligand on B cells, potentially disrupting the interaction between B cells and T_RM_ cells, which is critical for a potent immune response. These findings suggest that while B cells play a central role in both cancer types, their influence on the CD161/CLEC2D interaction in OPSCC might counteract the benefits observed in gastric cancer immunotherapy. The contrasting roles of T_RM_ cells highlight the intricate nature of the TME and emphasize the need for tailored immunotherapy approaches. Interventions that modulate the B cell–T_RM_ cells axis could be key to improving immunotherapeutic outcomes. Future strategies might include therapies that harness the beneficial effects of T_RM_ cells in gastric cancer or methods to overcome their suppressive influence on HPV‐positive OPSCC.

## 4 T_RM_ CELLS IN DISEASES

**FIGURE 2:**
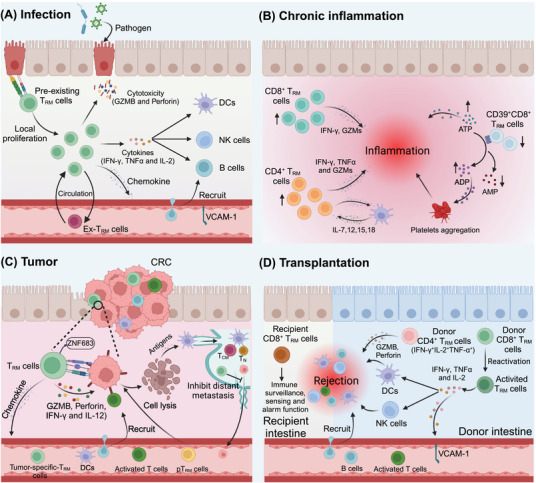
T_RM_ cells in diseases. (A) Infection: Preexisting T_RM_ cells respond quickly to reinfection by viruses or bacteria through local proliferation and attack infected cells with granzyme B and perforin. They release chemokines to recruit more immune cells to the infection site and secrete proinflammatory cytokines (IFN‐γ, TNF‐α, and IL‐2) to activate DCs, NK cells, and B cells. Some T_RM_ cells might leave the tissue, circulate, return, and transform back into T_RM_ cells. (B) Inflammatory bowel disease (IBD): In IBD, CD4^+^ T_RM_ cells are activated by cytokines (IL‐7, 12, 15, 18), and the populations of CD4^+^ and CD8^+^ T_RM_ cells are increased in the gut mucosa. These cells produce inflammatory cytokines and cytotoxic granules, which contribute to inflammation. Conversely, the number of CD39^+^CD8^+^ T_RM_ cells is decreased in IBD patients, resulting in inflammation due to the accumulation of ATP and ADP. (C) Colorectal cancer (CRC): In CRC, cancer‐specific T_RM_ cells marked by ZNF683 kill tumor cells by producing GZMB, and the released tumor antigens can prime new tumor‐specific T cells. T_RM_ cells also produce perforin, IFN‐γ, and IL‐2 to inhibit tumor growth and secrete chemokines, which can recruit DCs, activated T cells, and other immune cells to the TME. Antigen uptake from dying tumor cells by DCs can prime naïve T cells (T_N_ cells) or T_CM_ cells in draining lymph nodes, generating new cells (pT_RM_ cells) to sustain the cancer immune cycle. These cells can prevent distant metastasis by priming new cells in the tumor‐draining lymph nodes. (D) Transplantation: In intestinal transplants, T_RM_ cells from donors or recipients can sense and respond to stimuli by producing cytokines (IFN‐γ, TNF‐α, and IL‐2), causing inflammation and immune cell recruitment. Activated T_RM_ cells, especially Th1 cells, can lead to immune rejection if they produce Gzm B and perforin. In patients without rejection, donor T cells are gradually replaced by recipient T cells. This figure was designed using BioRender (https://biorender.com/).

TRM cells have been implicated in various tissues and associated diseases. They are found in most human tissues, including the brain, lung, liver, gastrointestinal tract, bone marrow, urogenital tract, skin, kidney, lymphoid tissues, and spleen, and they often have distinct antigen‐targeting profiles.^84^, ^85^, ^86^, ^87^, ^88^, ^89^, ^90^, ^91^, ^92^, ^93^ Their specific locations within tissues enable them to swiftly produce localized effector responses following a secondary challenge, thereby ensuring more rapid protection than other cell types do.^70^ T_RM_ cells play crucial roles in infectious diseases, tumors, chronic inflammatory conditions, and graft‐versus‐host disease, which we discuss in detail below (Figure 2).

### 4.1 T_RM_ cells in cancer

The TME exhibits highly complex heterogeneity among different types of tumors, and a variety of immune cells are present in tumors, including T cells, B cells, TAMs, neutrophils, DCs, myeloid‐derived suppressor cells, and natural killer (NK) cells.^94^, ^95^ T_RM_ cells include CD4^+^ T_RM_ cells and CD8^+^ T_RM_ cells. CD4^+^ T_RM_ cells respond mainly to viruses, parasites, and fungal pathogens, whereas CD8^+^ T_RM_ cells play crucial roles in tumor immune surveillance and immunotherapy.^96^, ^97^ In addition, the intratumoral infiltration of CD8^+^ T_RM_ cells is closely related to favorable prognostic outcomes in patients with cancer.^98^ Tumor‐localized CD8^+^ T_RM_ cells in solid tumors are frequently defined by the expression of CD103, CD69, and/or VLA‐1 (CD49).^99^, ^100^ Over the past decade, T_RM_ cells expressing CD69 or CD103 have been identified in multiple human solid tumors, including glioblastoma (GBM), hepatocellular carcinoma (HCC), esophageal squamous cell carcinoma (ESCC), gastric cancer (GC), colorectal cancer (CRC), intrahepatic cholangiocarcinoma (ICC), pancreatic ductal adenocarcinoma (PDAC), NSCLC, nasopharyngeal carcinoma (NPC), laryngeal squamous cell carcinoma (LSCC), triple‐negative breast cancer (TNBC), bladder cancer, cervical cancer, endometrial cancer, head and neck cancer (HNSC), melanoma, and urogenital tumors.^101^, ^102^, ^103^, ^104^, ^105^, ^106^, ^107^, ^108^, ^109^, ^110^, ^111^, ^112^, ^113^, ^114^, ^115^, ^116^

#### 4.1.1 T_RM_ cells drive antitumor immunity

The evidence indicates that T_RM_ cells play a potent role in antitumor immunity. Preclinical models support the notion that T_RM_ cells play a central role in tumor immunosurveillance.^117^ The upregulation of CD103, CD69, and CD49a strengthens the ability of T_RM_ cells to settle in the tumor niche, improving their capacity to fight tumors.^36^, ^43^, ^46^ Despite their cytotoxic activity, T_RM_ cells release significant amounts of cytokines and chemokines, particularly IFN‐γ and TNF‐α, which can activate other immune cells with antitumor potential. IFN‐γ produced by T_RM_ cells promotes the production of chemokines and antimicrobial molecules within the TME and recruits circulating T_CM_ cells.^111^, ^118^ Importantly, circulating T_CM_ cells have been shown to differentiate into T_RM_ cells in tumor models.^119^ TNF‐α released by T_RM_ cells in the TME can promote rapid DC maturation and upregulate the lymph node‐homing chemokine receptor CCR7.^120^ In addition, IL‐2 produced by T_RM_ cells in viral infection models has been shown to promote the upregulation of granzyme B (GZMB) in NK cells.^120^ Recently, virus‐specific T_RM_ cells (CD103^+^CD69^+^) for influenza A virus (IAV), Epstein–Barr virus (EBV), and/or cytomegalovirus (CMV) were reported to populate the mouse and human GBM microenvironments, and hepatitis B virus (HBV)‐specific CD8^+^ T_RM_ cells in HCC tissues can be reactivated by viral peptides.^102^, ^121^ Their reactivation by the local injection of viral peptides triggers rapid proliferation of T_RM_ cells and induces the production of cytokines, such as IFN‐γ, TNF‐α, and IL‐2.^121^, ^122^ Furthermore, the injection of viral peptides can induce antitumor immune activation, leading to the accumulation of CD8^+^ T cells, NK cells, and DCs within tumors, activating DCs in the draining lymph nodes (LNs), and limiting tumor growth.^80^, ^123^ Within the CD8^+^ T_RM_ cell population, the presence of two distinct subsets, TCF1^+^ and TCF1^−^, further underscores the complexity of T_RM_ cell function in melanoma.^124^ TCF1^+^ T_RM_ cells exhibit increased proliferative and effector potential, characterized by elevated expression of IFN‐γ and Ki67. These cells appear to function as progenitor‐like cells within the TME, facilitating the expansion and differentiation of effector T_RM_ cells, including TCF1^−^ T_RM_ cells. Conversely, TCF1^−^ T_RM_ cells exhibit a more cytotoxic phenotype and are closely associated with apoptotic melanoma cells. These cells directly recognize and eliminate tumor cells, playing a crucial role in tumor clearance. These findings indicate that antiviral T_RM_ cells can be reactivated within tumors, initiating an immune response.

Within the TME, nutrient depletion and excessive production of metabolic byproducts by tumors disrupt T‐cell metabolic and epigenetic programs, thereby impairing T‐cell antitumor immunity. Hypoxia is a common feature of most solid tumors and is generally associated with reduced T‐cell functionality. In the TME, hypoxia triggers the expression of hypoxia‐inducible factor 1‐alpha (HIF‐1α), leading to metabolic changes that ultimately weaken the antitumor activity of T cells.^125^ In contrast, HIF‐1α and HIF‐2α activity actually enhances the antitumor effector functions and tissue residency of T_RM_ cells by promoting the production of IFN‐γ and the expression of the integrin CD103. In the B16 tumor model, CD8^+^ tumor‐infiltrating lymphocytes (TILs) deficient in von Hippel–Lindau, a negative regulator of HIF, exhibited a gene expression profile typical of T_RM_ cells. This profile was closely linked to an exhaustion signature, suggesting that these cells retained effector function and were responsive to checkpoint blockade. As a result, this memory subset was able to mediate the complete regression of established B16 melanoma tumors.^126^ In contrast to T_RM_ cells in other tissues, lung T_RM_ cells have a shorter lifespan and require repeated antigenic stimulation to be maintained, which limits their ability to provide long‐term protection against respiratory diseases.^69^ While the exact mechanism behind this difference remains unclear, it may be partially influenced by factors such as oxygen levels in the lung environment. Despite hypoxia, fatty acid oxidation plays a critical role in the maintenance and function of T_RM_ cells. When deprived of fatty acids, T_RM_ cells undergo cell death.^127^ The purinergic receptor P2Rx7 is highly expressed in many T_RM_ cell populations, promoting metabolic fitness and cell survival.^128^ Additionally, the upregulation of the mevalonate‐cholesterol metabolic pathway promotes the formation of T_RM_ cells and enhances the antitumor immune effect of T_RM_ cells within the TME.^129^ These findings suggest that leveraging the metabolic programming utilized by T_RM_ cells could promote the survival and function of CD8^+^ TILs within the TME.

#### 4.1.2 Inhibitory checkpoint molecules expressed on T_RM_ cells within the TME

Substantial evidence has shown that a greater density of CD103^+^CD8^+^ T_RM_ cells in the TME is associated with a better response to immunotherapy and a better survival outcome for patients with cancer.^35^, ^36^, ^99^, ^107^, ^130^, ^131^ In patients with NSCLC, the density of T_RM_ cells (CD103^+^CD8^+^) is significantly correlated with the expression of PD‐1, Tim‐3, and GZMB and is negatively associated with tumor angiogenesis.^108^, ^132^, ^133^ CD103^+^CD4^+^ PD‐1^low^ TILs produce the most effector cytokines (TNF‐α and IFN‐γ), and CD103^+^CD4^+^PD‐1^+^ and CD69^+^CD4^+^PD‐1^+^ TILs produce CXCL13.^134^ In vivo and in vitro, PD‐L1 blockade can increase the proportion of CD8^+^CD103^+^ T_RM_ cells in GC tissues and restore the cytolytic capacity by increasing fatty acid binding protein (Fabp) 4 and Fabp5 expression.^104^, ^135^ Single‐cell sequencing revealed that CD8^+^ T_RM_ cells exhibit an effector memory phenotype (CD103‐, CD49α‐, GZMB‐ and CXCL13‐expressing) and exhaustion markers (CTLA‐4‐, TIM‐3‐, PD‐1‐ and TIGIT‐expressing) in GC tissues.^136^ Interestingly, these CD103^+^CD69^+^CD8^+^ T_RM_ cells can be suppressed by the gastric microbiome, particularly *Methylobacterium*, leading to tumor immune escape and a poor prognosis for patients with GC.^136^ In CRC and CRC liver metastasis patients, the infiltration and IFN‐γ secretion of these T_RM_ cells can be increased by antiangiogenic therapy, and infusing CD103^+^CD8^+^ T_RM_ cells back into the body can achieve better antitumor effects.^137^ Recent research has identified zinc finger protein 683 (ZNF683) expression as a candidate marker of cancer‐specific T_RM_ cells in CRC.^105^ These findings indicate that ZNF683 is a promising target for the regulation of cancer immunity. Muscle‐invasive bladder cancer (MIBC) patients with high infiltration of CD103^+^CD8^+^ T_RM_ cells, rather than those with infiltration of CD8^+^ T cells alone, are more likely to benefit from both immunotherapy and adjuvant chemotherapy (ACT). This is especially applicable in patients with proficient mismatch repair, homologous recombination, or activation of the PIK3CA/AKT and RAS/RAF pathways or patients who are deficient in histone modification and expression of markers related to cell cycle pathways.^138^ In patients with TNBC (a typically cold tumor), CD8^+^CD103^+^ T_RM_ cells are particularly important for immunotherapy and tend to express higher levels of immune checkpoint molecules, such as PD‐1, CTLA‐4, TIM‐3, and LAG3.^139^ Moreover, the clonal overlap of T_RM_ cells in breast cancer tissues with those in the urogenital tract may indicate a shared mechanism in the immune response in which T_RM_ cells traffic between tissues and contribute to coordinated defense against malignancies.^36^, ^140^ This systemic connection could be a key factor in developing multimodal cancer therapies that harness the power of T_RM_ cells across different compartments of the body.

These findings suggest that CD8^+^ T_RM_ cells are a prognostic biomarker of survival in patients with tumors and could help identify patients who are likely to benefit from immunotherapy. Inhibitory checkpoint molecules expressed on T_RM_ cells may serve as potential targets for cancer immunotherapy.

#### 4.1.3 Complexity of T_RM_ cells within the TME

The presence of T_RM_ cells in the TME is not always beneficial. In certain contexts, T_RM_ cells can also perform immunosuppressive functions, potentially promoting tumor growth and metastasis. For example, in cutaneous squamous cell carcinoma (cSCC), CD8^+^CD103^+^ T_RM_ cells display an immunosuppressive phenotype and are associated with poor prognosis.^141^ These cells highly express immunosuppressive markers such as CD39, CTLA‐4, and PD‐1 and produce relatively high amounts of IL‐10, which inhibits antitumor immune responses and promotes tumor growth and metastasis. Furthermore, CD8^+^CD103^+^ T_RM_ cells are more abundant in cSCC tissues than in normal skin and blood, suggesting their involvement in tumor immune surveillance or immune evasion. Negative correlations between T_RM_ cells and patient survival have also been reported in metastatic clear‐cell renal cell carcinoma (ccRCC) and GBM.^142^, ^143^ These findings underscore the complex role of T_RM_ cells in the tumor immune microenvironment. Therefore, a comprehensive understanding of the complex functions of T_RM_ cells is crucial for the development of more effective treatments for tumors. To better understand the characteristics of T_RM_ cells in various types of tumors, we compiled a table showing the distribution and properties of these cells across different tumor types (Table 2).

**TABLE 2:** The features of T_RM_ cells in different types of human tumors.

Tumor position	Tumor type	T_RM_ cell phenotype	Inhibitory receptors	Main findings	Cytokines	References
CNS	GBM	CD8^+^CD103^+^	PD1, TIM3	CD8^+^CD103^+^ T_RM_ cells are crucial in GBM, with low PD1 and TIM3 levels correlating with better OS and PFS. Blocking PD1 and TIM3 enhances their antitumor activity.	GZMB, IFN‐γ, TNF‐α	^211^
		CD8^+^CD69^+^CD103^+^CD49a^+^	PD1, LAG3, TIM3, CTLA4	In GBM, CD8^+^ T_RM_ cells’ function is suppressed by inhibitory receptors, downregulated functional molecules, and exhaustion‐related transcription factors such as NR4A1, MAF, and IRF4.	GZMB, Perforin 1, IFN‐γ, TNF‐α	^212^
		CD8^+^CD69^+^CD103^+^	‐	High CD103 expression and abundant CD8^+^ TILs are associated with longer survival, while high CD103 with low CD8^+^ TILs correlates with poorer outcomes.	‐	^213^
	Glioma	CD8^+^CD69^+^CD103^+^/CD8^+^CD69^+^TCF1^+^	PD‐1, TIGIT, LAG3	In pediatric gliomas, two T_RM_ subsets exist: TCF1^+^ T_RM_ cells near blood vessels and CD103^+^ T_RM_ cells within the tumor, showing spatial heterogeneity. Recurrent tumors lose CD103^+^ T_RM_ cells and gain TCF1^+^ T_RM_ cells, which are linked to angiogenesis.	GZMB	^214^
		CD45RO^+^CCR7^−^CD69^+^CD4^+^/CD45RO^+^CCR7^−^CD69^+^CD103^+^CD8^+^	PD‐1, TIGIT, PD‐L1,	In pediatric and young adult gliomas, T_RM_ cells with the CD45RO^+^CD69^+^CCR7^−^ phenotype are present in tumor tissues and are linked to better patient prognosis.	‐	^101^
RT	HNSCC	CD8^+^CD103^+^/CD8^+^CD103^−^	TIM3	In HNSCC, T_RM_ cells are linked to better prognosis in primary tumors but not in metastatic tumors. Both primary and recurrent tumors contain T_RM_ cells with high TIM3 expression, unlike lymph node metastases.	‐	^215^
	OSCC	CD45RO^+^CCR7^−^CD69^+^CD103^+^CD8^+^	PD‐1^low^	An increased density of CD103^+^CD8^+^ T_RM_ cells in the stroma and CD103^+^CD11c^+^ TILs within the tumor indicates a favorable prognosis in OSCC.	‐	^216^
	NSCLC	CD103^+^CD69^+^CD8^+^/CD69^+^CD8^+^	PD‐1	In NSCLC, tumor‐associated CD8^+^ T_RM_ cells express JAML, which binds to the cancer‐derived CXADR, activating the T_RM_ cells. Higher numbers of CD8^+^ T_RM_ cells and JAML expression are positively correlated with better patient prognosis.	GZMB, NCR1, IFN‐γ, GZMA, MZB1, CXCL10, CXCL13, CXCR5	^217^
		CD103^+^CD8^+^/CD103^+^CD4^+^	PD‐1	In resectable NSCLC, neoadjuvant chemotherapy enhances antitumor immunity by recruiting T and B cells and promoting a shift toward cytotoxic and memory CD8^+^ and CD4^+^ helper T cells.	‐	^20^, ^218^
		CD103^+^CD8^+^	PD‐1, TIM‐3	In NSCLC, four T_RM_ cell subgroups were identified: PD‐1^−^Tim‐3^−^T_RM_ cells, PD‐1^+^Tim‐3^−^ T_RM_ cells, PD‐1^−^Tim‐3^+^ T_RM_ cells, and PD‐1^+^Tim‐3^+^ T_RM_ cells. Among these, PD‐1^+^Tim‐3^−^ T_RM_ cells exhibited the highest level of cytotoxicity.	GZMB	^108^
	NPC	CD103^+^CD8^+^	‐	In NPC, CD103^+^CD8^+^ T_RM_ cells in cancer islets are a key CD8+ TIL subset that, through elevated IL‐17 expression, may indicate a higher risk of local recurrence.	IL‐17	^109^
	LSCC	CD103^+^CD8^+^	PD‐L1	In recurrent LSCC, a high presence of CD103^+^ TILs and elevated PD‐L1 levels are associated with significantly improved OS, DSS, and DFS.	‐	^219^
DT	ESCC	CD103^+^CD69^+^CD8^+^	PD‐1, TIM‐3, LAG‐3, TIGIT	In ESCC patients, CD103^+^CD8^+^ T_RM_ cells are enriched in tumor tissues and metastatic lymph nodes, correlating with improved OS, controlled lymphatic invasion, and reduced metastasis.	GZMB, IFN‐γ, IL‐2, LAMP‐1	^103^, ^220^
	GC	CXCL13^+^CD103^+^CD8^+^	PD‐1	B cells in tertiary lymphoid structures promote glycolysis in CXCL13^+^CD103^+^CD8^+^ T_RM_ cells via the TNFR2 axis, boosting the efficacy of anti‐PD‐1 therapy.	GZMB, IFN‐γ,	^73^
		CD103^+^CD8^+^	PD‐1^high^, 4‐1BB^high^	In gastric cancer, the number of tumor‐infiltrating CD8^+^CD103^+^ T_RM_ cells is reduced, their cytotoxic function is impaired, and this is negatively correlated with cancer progression and survival. Combining PD‐1 blockade with 4‐1BB costimulation can restore their function.	‐	^104^
	CRC	CD103^+^CD8^+^	PD1, CTLA4, ICOS	The higher the number of CD103^+^CD8^+^ TILs in the tumor tissue of CRC patients, the longer their OS and RFS. Additionally, the study identified ZNF683 as a candidate marker for tumor‐specific T_RM_ cells.	GZMA, GZMB, IFN‐γ	^105^
		CD103^+^CD8^+^	‐	In CRC patients, infiltration of CD103^+^CD8^+^ T_RM_ cells is positively correlated with earlier clinical stages and negative VEGF expression, indicating a better prognosis and showing potential predictive value for liver metastasis development.	‐	^137^
	HCC	CD103^+^CD38^+^CD8^+^	PD‐1, CTLA4, TIM‐3, LAG3 TIGIT	CD38 is a marker of exhausted CD8^+^ T_RM_ cells in the HCC TME, and its coexpression with PD‐1 suggests its role in disease aggressiveness.	‐	^221^
		CD103^+^CD8^+^	PD‐1	HBV infection alters the immune microenvironment at the tumor margins in HCC patients, increasing PD‐1^+^CD8^+^ T_RM_ cells with impaired function, which exacerbates HBV‐related liver damage and fibrosis.	IFN‐γ	^222^
		CD103^+^CD69^+^CD8^+^	PD‐1^high^	CD103^+^ T_RM_ cells expressing high levels of PD‐1 in the HCC TME are associated with better patient survival, in contrast to PD‐1^+^‐exhausted T cells, which are linked to poor progression‐free survival.	CXCR6	^223^
	CCA	CD103^+^CD39^+^CD69^+^CD8^+^	TIM‐3, PD‐L1, PD‐1^high^, 4‐1BB, GITR	CCA with a higher proportion of CD69^+^CD103^+^CD8^+^ T_RM_ cells showed a greater number of TIL infiltrates, higher PD‐L1 expression on the tumor, and higher expression levels of the T‐cell‐inflamed gene signature.	‐	^224^
	PDAC	CD103^+^CD39^+^CD8^+^	PD‐1, TIGIT	T_RM_ cells coexpressing PD‐1 and TIGIT in PDAC tumors are associated with better prognosis; tumor infiltration by T_RM_ cells is linked to an enhanced response to immunotherapy.	IFN‐γ, CCL4	^107^, ^225^
		CD103^+^CD39^+^CD8^+^	‐	In PDAC tumor tissues, CD103^+^CD8^+^ T_RM_ cells are significantly reduced and lack activation markers and PD‐1 expression. However, a new T_RM_ subset, CD127^–^CD103^+^CD39^+^CD45RO^+^ ILC1‐like cells, is enriched.	‐	^226^
		CD103^+^CD39^+^CD8^+^	PD‐1	A lower proportion of CD103^+^PD‐1^+^CD39^+^ T_RM_ cells in the PDAC TME is associated with poorer prognosis and a higher risk of liver metastasis.	‐	^227^
UT	EC	CD103^+^CD39^+^CD8^+^	PD‐1, CTLA4, TIM‐3, LAG3 TIGIT	CD39^+^CD103^+^CD8^+^ T_RM_ cells in high‐grade EC retain a polyfunctional, activation‐responsive repertoire even in an exhausted state.	IFN‐γ, TNF‐α, IL‐2, IL‐21, GM‐CSF	^228^
		CD103^+^CD8^+^	PD‐1, TIGIT	PD‐1 and TIGIT are significantly coexpressed in T_RM_ cells within the EC TEM, which display an exhausted phenotype with impaired cytotoxicity, increased proliferation, and reduced cytotoxic activity.	GZMA, GZMB, GZMH,	^229^
	CC	CD103^+^CD8^+^	‐	In CC tissues, higher CXCL13^+^CD8^+^ T_RM_ cells abundance and lower PLAC8^+^CD8^+^ T_RM_ cells abundance are positively correlated with OS and PFS after radiotherapy.	CXCL13,	^230^
	OC	CD103^+^CD69^+^CD49a^+^CD8^+^	PD‐1, CTLA4, TIM‐3, LAG3 TIGIT	The hallmarks of tumor recognition in OC‐infiltrating T cells are primarily dependent on T_RM_ cells, and only progenitor (TCF1^low^) T_RM_ cell stem cells can predict the prognosis of OC.	IFN‐γ, GZMB, CXCL13, CXCR6	^231^
	PC	CD103^+^CD8^+^	PD‐1, CTLA4	Patients with high levels of CD103^+^CD8^+^ T_RM_ cells in PC have a higher risk of biochemical recurrence and lower biochemical recurrence‐free survival, indicating a poorer prognosis.	IL‐10	^232^
	RCC	CD103^+^CD69^+^CCD8^+^	PD‐1,	The CD69^+^CD103^+^CD8^+^ T_RM_ cells in RCC tissue express higher levels of HLA‐DR and PD‐1 and lower levels of CD28. These cells are potentially associated with kidney transplantation and kidney infections.	IL‐2, IL‐17, TNF‐α, GZMB	^233^
	BC	CD103^+^CD8^+^	‐	Patients with higher infiltration of CD103^+^CD8^+^ T_RM_ cells in muscle‐invasive BC have longer survival and are more likely to benefit from immunotherapy and adjuvant chemotherapy.	‐	^138^
	TNBC	CD103^+^CD39^+^CD69^+^CD8^+^	PD‐1, TOX, LAG3 TIGIT	CD39^+^ T_RM_ cells are enriched in TNBC, are associated with longer patient survival, and can serve as a biomarker for immune checkpoint inhibitors treatment response.	CXCL13, IFN‐γ, GZMB	^234^
		CD103^+^CD8^+^	PD‐1, CTLA4, TIM‐3, LAG3 TIGIT	CD103^+^CD8^+^ T_RM_ cells are enriched in the TME of TNBC and are associated with improved DFS or OS in patients.	GZMB, perforin	^111^
Skin	Melanoma	CD103^+^CD8^+^	PD‐1	TCF1^+^ and TCF1^−^ T_RM_ cells, as well as cDC1, play a crucial role in melanoma control and response to checkpoint blockade immunotherapy.	IFN‐γ, Ki67	^124^
		CD103^+^CD8^+^	‐	In melanoma patients, higher CD8^+^ T_RM_ cell infiltration is positively associated with T cells, NK cells, M1 macrophages, and memory B cells in the TME and correlates with longer overall survival.	‐	^235^
	CSCC	CD103^+^CD69^+^CD39^+^CD8^+^	PD‐1, CTLA4	CD103^+^CD8^+^ T_RM_ cells in CSCC exhibit dysfunctional features, are associated with poorer clinical outcomes, and are more common in metastatic CSCC.	IL‐10	^141^

Abbreviations: BC, bladder cancer; CC, cervical cancer; CCA, cholangiocarcinoma; CCL4, chemokine (C‐C motif) ligand 4; cDC1, classic dendritic cell type 1; CNS, central nervous system; CRC, colorectal cancer; CSCC, cutaneous squamous cell cancer; CTLA4, cytotoxic T‐lymphocyte‐associated protein 4; CXCL10, chemokine (C‐X‐C motif) ligand 10; CXCL13, chemokine (C‐X‐C motif) ligand 13; CXCR5, chemokine (C‐X‐C motif) receptor 5; CXCR6, chemokine (C‐X‐C motif) receptor 6; DFS, disease‐free survival; DSS, disease‐specific survival; DT, digestive tract; EC, endometrial cancer; ESCC, esophageal squamous cell carcinoma; GBM, glioblastoma; GC, gastric cancer; GITR, glucocorticoid‐induced TNF receptor; GM‐CSF, granulocyte‐macrophage colony‐stimulating factor; GZMA, granzyme A; GZMB, granzyme B; GZMH, granzyme H; HBV, hepatitis B virus; HCC, hepatocellular carcinoma; HNSCC, head and neck squamous cell carcinoma; IFN‐γ, interferon gamma; IL‐10, interleukin‐10; IL‐17, interleukin‐17; IL‐2, interleukin‐2; IL‐21, interleukin‐21; IRF4, Interferon Regulatory Factor 4; LAG3, lymphocyte activation gene 3; LAMP‐1, lysosome‐associated membrane protein 1; LSCC, laryngeal squamous cell carcinomas; MAF, microphthalmia‐associated transcription factor; MZB1, macrophage activation factor 1; NCR1, natural cytotoxicity receptor 1; NPC, nasopharyngeal carcinoma; NR4A1, nuclear receptor subfamily 4, group A, member 1; OC, ovarian cancer; OS, overall survival; OSCC, oral squamous cell carcinoma; PC, prostate cancer; PD1, programmed cell death protein 1; PDAC, pancreatic ductal adenocarcinoma; PD‐L1, programmed death‐ligand 1; PFS, progression‐free survival; RCC, renal cell carcinoma; RFS, recurrence‐free survival; RT, respiratory tract; TCF1, T‐cell factor 1; TCF1, transcription factor T‐cell factor 1; TIGIT, T‐cell immunoglobulin and ITIM domain; TILs, tumor‐infiltrating lymphocytes; TIM‐3, T‐cell immunoglobulin and mucin domain‐containing protein 3; TME, tumor microenvironment; TNBC, triple‐negative breast cancer; TNF‐α, tumor necrosis factor‐alpha; TOX, thymocyte selection‐associated high mobility group box protein; UT, urogenital tract; VEGF, vascular endothelial growth factor; ZNF683, zinc finger protein 683; ICOS, inducible T‐cell co‐stimulator.

### 4.2 T_RM_ cells in infections

#### 4.2.1 T_RM_ cells in virus infections

Many viruses such as JC polyomavirus, varicella zoster virus (VZV), herpes simplex virus (HSV), poliovirus, Zika virus, and West Nile virus (WNV) are neurotropic or gliatropic. They can induce neurological diseases such as meningitis, myelitis, encephalitis, and demyelination.^144^ The presence of T_RM_ cells is particularly necessary to combat recurrent infection by neurotropic viruses. In a mouse polyomavirus encephalitis model and in mouse cytomegalovirus, HSV, and WNV infection mouse models, CD8^+^ T_RM_ cells play an important role in controlling viral infections by secreting effector molecules such as granzymes, perforin, and IFN‐γ, which persist in the central nervous system (CNS) parenchyma after the clearance of viral infections.^145^, ^146^, ^147^ The increase in antigen‐specific CD8^+^ T_RM_ cells in the CNS limits brain infections. Importantly, the CXCL10/CXCR3 chemokine pathway, which protects against recurrent HSV‐1 infection and disease, is critical in shaping T_EM_ and T_RM_ immunity in a murine model.^147^

During IAV infection, CD8^+^ T_RM_ cells are recruited from the lung to the airways through the CXCR6/CXCL16 signaling axis to achieve ectopic defense.^148^ These T_RM_ cells are vital for protection against IAV reinfection and can produce large quantities of cytokines, such as IFN‐γ, TNF‐α, perforin, and GZMB, which confer this protective function.^149^, ^150^ Moreover, CD4^+^ resident helper T (T_RH_) cells play a critical role in the formation of lung CD103^+^CD8^+^ T_RM_ cells during IAV infection by secreting IFN‐γ and limiting the expression of the transcription factor T‐bet.^151^ This promotes the development of protective B‐cell and CD8^+^ T_RM_ cell responses after the resolution of primary IAV infection, as well as during RSV infection.^152^, ^153^ Notably, optimal protection against SARS‐CoV infection in mice is provided by airway memory CD4^+^ T cells that secrete proinflammatory IFN‐γ and the anti‐inflammatory cytokine IL‐10.^154^ CD4^+^ T_RM_ cells can also play direct protective roles during subsequent viral encounters in an IFN‐γ‐dependent manner.^155^ These findings indicate that CD4^+^ T_RM_ cells contribute to both direct and indirect defense mechanisms against virus infections. Several human studies have reported the detection of CD4^+^ T_RM_ cells and CD8^+^ T_RM_ cells within the female reproductive tract after infection with HSV‐2.^156^, ^157^ In the early stages of HSV‐2 infection in mice, virus‐specific CD4^+^ T cells rapidly infiltrate vaginal tissues, followed by a significant influx of virus‐specific CD8^+^ T cells.^158^ Remarkably, these T_RM_ cells are maintained independently of IL‐15 expression, suggesting a unique homeostatic mechanism that is finely attuned to the local environment.^159^ The severity of HSV‐2 infection may be influenced by the density of these T_RM_ cells at a specific infection site, where they primarily rely on cytokine secretion (notably that of IFN‐γ) for their antiviral effects rather than on their ability to clear the virus via cytotoxic mechanisms.

Together, these data indicate that both CD4^+^ T_RM_ cells and CD8^+^ T_RM_ cells play important roles in local antiviral immunity.

#### 4.2.2 T_RM_ cells in bacterial infections

CD4^+^ T_RM_ cells play an important role in mediating the body's immune response against bacteria. In a mouse model infected with mismatched serotypes of *Streptococcus pneumoniae*, prior infection led to the seeding of CD4^+^ T_RM_ cells in previously infected lung lobes. Upon stimulation with heterotypic *S. pneumoniae*, these cells produce multiple effector cytokines (notably IL‐17A) and promote neutrophil recruitment in the lungs.^160^ Researchers have also reported that in heat‐killed *Klebsiella pneumoniae*‐immunized IL‐17A tracking‐fate mouse models, lung CD4^+^ T_RM_ cells are derived from IL‐17A‐producing effector (Th17) cells and are maintained in the lungs by IL‐7.^161^ During *Staphylococcus aureus* infection, these T_RM_ cells rapidly proliferate and produce proinflammatory cytokines, such as IL‐17A and IL‐22, which contribute to the clearance of the pathogen and protection against future infections.^162^ Similar results were reported in *Candida albicans* skin infections, which suggested that lung CD4^+^ T_RM_ cells play an important role in bacterial clearance.^163^ Furthermore, IL‐17‐producing CD4^+^ T_RM_ cells persist long‐term in the colonized oral mucosa and play a crucial role in maintaining homeostasis and preventing overgrowth of commensal fungi.^164^

During acute *Heliobacter pylori (H. pylori)* colonization in both mice and humans, CD8^+^ T_RM_ cells infiltrate the gastric mucosa in response to encountering cytotoxin‐associated gene A (CagA), thereby contributing to pathogen control and pathology.^165^ In addition, *H. pylori* vaccine‐induced CD4^+^ T_RM_ cells (CD69^+^CD103^−^) can proliferate and differentiate into effector Th1 cells in situ after *H. pylori* challenge to enhance gastric local immunity during the recall response.^166^ In vivo experiments in mice have shown that neuroinvasive *Listeria monocytogenes* infection induces the accumulation of CD8^+^ T_RM_ cells in the brain and that the expression of miR‐155 is necessary for the optimal accumulation of CD8^+^ T_RM_ cells.^167^ In an *Eimeria vermiformis* infection model in the small intestine, type 1 Treg cells are selectively drawn to local inflammatory sites through the chemokine receptor CXCR3 and promote the generation of CD8^+^ T_RM_ cells by expressing integrin‐β8.^168^

However, recent studies have shown the potential for CNS T_RM_ cells to act in a deleterious manner upon antigen‐specific reactivation. Antigen‐specific T_RM_ cells can induce significant neuroinflammation, neuropathology, and peripheral immunosuppression.^169^, ^170^, ^171^ Virally induced CD8^+^ T_RM_ cells have been shown to cause cognitive decline, mainly through IFN‐γ stimulation of microglia.^172^ Reducing the number of CD8^+^ T_RM_ cells could be a novel strategy for mitigating long‐term brain inflammation. Therefore, balancing the role of CD8^+^ T_RM_ cells in brain infections requires precise immune regulation to ensure effective anti‐infection responses while preventing excessive inflammatory damage.

#### 4.2.3 T_RM_ cells in parasite infections

In addition to their roles in viral and bacterial infections, T_RM_ cells also participate in combating parasitic infections by secreting cytokines. GATA3^+^CD4^+^ Th2 memory cells persist in the peritoneal cavity and small intestinal lamina propria after natural murine infection with *Heligmosomoides polygyrus*. These T_RM_ cells proliferate in situ and express IL‐4, IL‐5, and IL‐13 to mediate anthelminthic effects.^173^ In C57BL/6 mice infected with *Schistosoma japonicum*, there were significant changes in the content, memory‐related molecule expression, and cytokine production of pulmonary CD4^+^CD103^+^ T cells and CD8^+^CD103^+^ T cells. These findings demonstrate that pulmonary T_RM_ cells play important roles in mediating granulomatous inflammation induced by *S. japonicum* infection.^174^ *Leishmania*‐specific CD4^+^ T_RM_ cells produce IFN‐γ, resulting in protection against reinfection with *L. major*.^175^ In the context of chronic *Toxoplasma gondii* infection, CD8^+^ T_RM_ cells accumulate within the brain and possess the inherent ability to produce both IFN‐γ and TNF‐α, which are crucial for controlling the parasite within the CNS.^176^

### 4.3 T_RM_ cells in chronic inflammatory diseases

Chronic inflammatory diseases can affect any organ or tissue in the body, including the central nervous system, gut, skin, joints, and muscles. These conditions include chronic virus infection, autoimmune conditions, and neurodegenerative disorders.^177^, ^178^ CD4^+^ and CD8^+^ T_RM_ (sub) cell populations are enriched in inflammatory diseases, and T_RM_ cell infiltration in tissues positively correlates with chronic inflammatory disease activity.^60^

CD4^+^ T cells play a critical role in maintaining the long‐term presence of CD8^+^ T_RM_ cells in the CNS by supporting their cytotoxic functions and contributing to progressive autoimmune‐driven neuronal damage.^169^, ^170^ A population of CD8^+^ T lymphocytes (CD69^+^CD122^−^PD1^+^CD44^+^CD62L^−^) uniquely accumulates in the CNSs of neuropsychiatric lupus‐prone mice.^179^ In humans, the population of T_RM_ cells (CD69^+^CD103^+^CD8^+^) is increased in the cerebrospinal fluid of patients with chronic inflammatory diseases, including multiple sclerosis, as well as neurodegenerative diseases, such as Alzheimer's disease and Parkinson's disease, compared with controls.^180^ T_RM_ cells from aged patients promote inflammation following ischemic stroke.^181^ These findings indicate that T_RM_ cells significantly influence the immunopathology of various chronic inflammatory processes in the CNS, potentially contributing to neurological decline. CD103^+^CD161^+^CCR5^+^CD4^+^ T_RM_ cells, which are specific to patients with Crohn's disease, produce significant amounts of type 1 inflammatory cytokines, thereby orchestrating the local inflammatory response.^182^ Additionally, CD4^+^ T_RM_ cells with a Th17 signature (as well as CD8^+^ T_RM_ cells) contribute to disease pathogenesis through IFN‐γ induction and subsequent chemokine production in myeloid cells.^183^ Highly activated CD8^+^ T_RM_ cells, which mediate bile duct damage and disease progression by producing IL‐17 and IL‐22, play important roles in primary sclerosing cholangitis and primary biliary cholangitis.^184^, ^185^ In chronic pancreatitis patients, the surface expression of PD‐1 was lower on CD8^+^ T_RM_ cells than on control pancreatic cells while T‐bet expression was increased, and the degree of T‐bet expression upregulation was significantly associated with PD‐1 expression downregulation.^186^ In chronic liver diseases caused by viral or parasitic infections, autoimmune hepatitis, and nonalcoholic steatohepatitis, the expansion of T_RM_ cells can result in heightened liver inflammation.^177^, ^187^, ^188^, ^189^, ^190^ Hopefully, the expansion of these T_RM_ cells can be directly inhibited by glucocorticoids, thereby reducing hepatic inflammation.^190^ Additionally, CD103 expression is observed on renal T cells, particularly on CD8^+^ T cells, in patients with systemic lupus erythematosus (SLE) and in SLE‐prone mice.^191^, ^192^ Infection with *S. aureus* or *C. albicans* causes kidney CD4^+^ T_RM_ cells to adopt an inflammatory Th17 phenotype, exacerbating the disease.^191^ The activation of kidney T_RM_ cells by cytokines such as IL‐1β, IL‐6, and IL‐23 through the JAK/STAT pathway amplifies the inflammatory response. The presence of these T_RM_ cells in chronic inflammatory diseases suggests a role for microbiota‐driven inflammation in the promotion of autoimmunity. In psoriasis patients with psoriatic arthritis and atopic dermatitis, T_RM_ cells, particularly IL‐17‐producing CD8^+^ T cells, contribute to the chronicity and relapse of the disease by producing cytokines such as IL‐4, IL‐13, IL‐17, and IL‐22.^193^, ^194^ T_RM_ cells are also involved in other chronic inflammatory skin diseases, including systemic sclerosis, cutaneous lupus erythematosus, frontal fibrosing alopecia, alopecia areata, polymorphic light eruption, allergic contact dermatitis, delayed‐type drug hypersensitivity, and fixed drug eruption.^195^, ^196^, ^197^, ^198^, ^199^, ^200^, ^201^ These cells contribute to the chronicity and recurrence of these diseases through similar mechanisms involving cytokine production and interactions with the skin barrier. Targeting T_RM_ cells and their proinflammatory cytokines, such as IL‐17 and IFN‐γ, may offer novel therapeutic approaches to prevent disease flares and promote long‐term remission.

However, the persistence of autoimmune diseases in the CNS and the specific role of T_RM_ cells in driving chronic autoimmunity remain underexplored. While CD8^+^CD103^+^ T_RM_ cells are clearly abundant in the CNS of patients with autoimmune conditions such as multiple sclerosis (MS), their precise contributions to lesion formation and chronic inflammation are still not fully understood.^171^ MS, for example, is a chronic inflammatory and immune‐mediated disease of the CNS characterized by the interaction of T and B cells, and both EBV‐infected B cells and EBV‐specific CD8^+^ T cells have been found in MS lesions.^202^ A subset of these CD8^+^ T cells, which resemble T_RM_ cells, express markers such as CD103, CD69, GZMB, and PD‐1. These T_RM_ cells may exhibit impaired control over EBV infection, potentially leading to sustained inflammation.^203^ In CNS autoimmune diseases, such as MS, T_RM_ cells not only persist but may amplify local inflammation. The inability of TRM cells to exit the tissue, combined with their enhanced proliferative capacity (indicated by markers such as CXCR6, Ki67, and GPR56), suggests that T_RM_ cells may contribute to the chronicity of inflammation in CNS autoimmunity. For example, an increase in CD69^+^CD103^+^CD49a^+^PD‐1^+^ T_RM_ cells has been observed in MS lesions, and this increase correlates with active inflammation and ongoing demyelination.^10^ Furthermore, T_RM_ cells may act as local reservoirs of inflammation, reactivating upon exposure to antigens or inflammatory signals in the tissue microenvironment. This is not only observed in MS but also may extend to other CNS autoimmune diseases, such as neuromyelitis optica and autoimmune encephalitis, emphasizing the potential pathogenic role of T_RM_ cells across various neuroinflammatory conditions.^204^, ^205^

### 4.4 T_RM_ cells in other diseases

A longitudinal analysis of blood and bronchoalveolar lavage fluid samples from more than 20 lung transplant recipients demonstrated that the long‐term persistence of donor lung T_RM_ cells (expressing CD69, CD103, and CD49a) is associated with a reduced incidence of clinical events that precipitate lung injury, such as primary graft dysfunction and acute cellular rejection.^206^ Kidney allografts contain donor‐derived T cells that predominantly express tissue residency‐associated markers such as CD69 and CD103, but it is still unclear how long donor‐derived cells persist in the graft. In kidney transplant rejection, allospecific T cells in recipients are recruited to the graft, where they develop into T_RM_ cells and damage the kidney through the production of cytokines (such as IFN‐γ, TNF‐α, and GZMB) and cytotoxic molecules.^207^ On the basis of these findings, we can conclude that a higher number of donor‐derived organ T_RM_ cells is associated with lower rates of organ transplant rejection.

In addition, skin‐resident regulatory T cells (Tregs) initially promote inflammation at the keratinocyte layer following barrier breach.^208^ In burn injuries, skin T‐cell populations shift from a resident phenotype to a circulating homing marker profile. The later stages of epithelial injury involve skin‐resident γδ‐T cells and BATF^+^CCR8^+^ skin Tregs, which are thought to promote physiological wound healing by participating in a tightly regulated response that includes pro‐ and anti‐inflammatory signals and growth factors.^209^, ^210^

## 5 POTENTIAL THERAPEUTIC STRATEGIES INVOLVING T_RM_ CELLS

**FIGURE 3:**
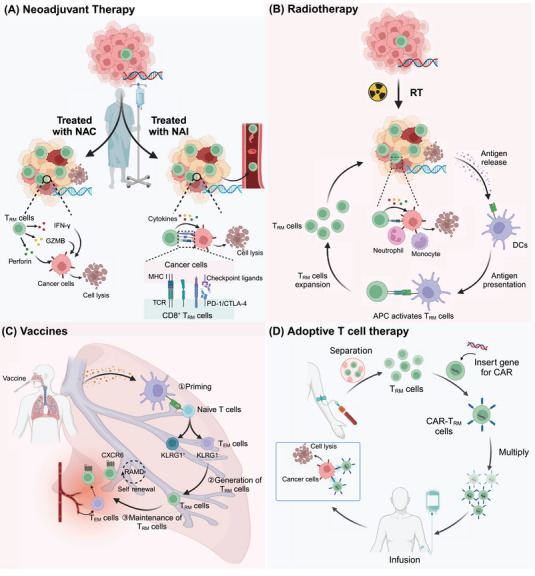
Potential therapeutic strategies based on T_RM_ cells. (A) Neoadjuvant therapy: (1) Neoadjuvant chemotherapy (NAC): Tumors treated with NAC can induce DNA chain breaks, leading to cell apoptosis. T_RM_ cells secrete IFN‐γ, GZMB, and perforin to kill tumor cells. (2) Neoadjuvant immunotherapy (NAI): Anti‐PD‐1/anti‐CTLA‐4 antibodies are used to block immune checkpoints and restore exhausted T_RM_ cells. Additionally, NAI can facilitate the exit of T_RM_ cells from tumors into the circulation, thereby promoting systemic tumor immunity. (B) Radiotherapy: T_RM_ cells exhibit strong radiation resistance and proliferate extensively when stimulated by tumor antigens. They secrete cytokines such as TGF‐β, which recruit neutrophils and monocytes into the tumor microenvironment, thereby promoting an antitumor immune response. (C) Vaccines: Vaccines induce the formation of pathogen‐specific T_RM_ cells in the lungs. The maintenance of lung CD8^+^ T_RM_ cells relies on replenishment from circulating CD8^+^ T_EM_ cells or on the local proliferation of lung CD8^+^ T_RM_ cells in repair‐associated memory depots (RAMDs). (D) Adoptive T‐cell therapy: T_RM_ cells from patients are collected and engineered to express a specific antigen on their surface, creating CAR‐T_RM_ cells. These CAR‐T_RM_ cells are then multiplied in large quantities in vitro and infused back into the patient's body to target and eliminate blood tumors. This figure was designed using BioRender (https://biorender.com/).

T_RM_ cells are found in various tissues in which they mediate powerful local innate and adaptive immune responses, offering long‐lasting protective immunity. They play crucial roles in protection against infectious and malignant diseases. In particular, the presence of T_RM_ cells is strongly associated with favorable clinical outcomes in patients. In addition, they are ideally positioned to act early in viral infections and prevent further spread. Therefore, increasing T_RM_ production or reactivating suppressed T_RM_ cells to increase treatment efficacy offers a highly promising therapeutic option for patients with tumors and other diseases (Figure 3, Table 3).

**TABLE 3:** Potential therapeutic strategies and clinical trials based on T_RM_ cells.

Therapeutic strategy	Disease type	Treatment strategy	Effects	References
Neoadjuvant chemotherapy	NSCLC	NCT + surgery	NCT was associated with a significant increase in CD8^+^ and CD4^+^ T_RM_ cells in the TME. These T_RM_ cells are involved in enhancing local antitumor immunity by promoting cytotoxic T‐cell responses and sustaining immune surveillance in resected tumors.	^218^
	EAC	NCT + surgery	Complete responders to NCT exhibited a higher frequency of CD39^+^CD103^+^CD8^+^ T_RM_ cells, which are associated with stronger antitumor immunity, potentially contributing to improved survival outcomes and a higher rate of pathologic complete response.	^281^
	HNSCC	NCT	In HNSCC patients, the density of intratumoral CD8^+^ T_RM_ cells was positively correlated with the efficacy of NCT, indicating that these cells may play a critical role in mediating tumor regression and improving treatment responses.	^282^
Vaccines	Infections	‐	This topic has already been covered in other reviews.	^263^
	HCC	Neoantigen peptide vaccine + α‐PD‐1	NeoVAC plus α‐PD‐1 could increase CD8^+^ T_RM_ cell infiltration in HCC TME.	^274^
	*Pseudomonas aeruginosa*	Intranasal vaccination with rePcrV	Intranasal vaccination with rePcrV induces the formation of CD4^+^ T_RM_ cells in the lungs, which helps resist *Pseudomonas aeruginosa*.	^283^
	Malaria	mRNA vaccine	mRNA‐based vaccine strategy to induce liver T_RM_ cells for the prevention of malaria.	^284^
	Pertussis	Nasal inoculation aPV/BcfA vaccine	Nasal administration of aPV/BcfA can generate Th17^+^CD4^+^ T_RM_ cells, effectively preventing the transmission and recurrence of pertussis.	^285^
	*Klebsiella pneumoniae*	outer membrane protein X and LTA1 adjuvant	The OmpX + LTA1 subunit vaccine generates *Klebsiella pneumoniae*‐specific Th1 and Th17 T_RM_ cells.	^286^
	LGGs	GBM6‐AD with poly‐ICLC	Neoadjuvant vaccination led to an enhancement of CD8^+^ T_RM_ cells exhibiting an effector memory phenotype within the TME.	^287^
	Breast cancer	Adenoviral vector vaccine	The adenoviral vector vaccine combined with IL‐1β induces the generation of pulmonary T_RM_ cells, reducing lung metastases in breast cancer.	^288^
CAR‐T‐cell therapy	Solid and liquid tumors	M5 CAR T cells	CAR‐T cells induced with TGF‐β acquired T_RM_ cell characteristics, enabling them to survive for a long time in the TME and rapidly eliminate cancer cells.	^289^
	PBC	PD‐1‐targeting CAR‐T cells	Treatment with PD‐1‐directed CAR‐T cells in DKO mice can selectively deplete hepatic CD8^+^ T_RM_ cells, thereby ameliorating autoimmune cholangitis.	^184^
	Solid tumor	CAR‐T cells overexpressing Runx3	Runx3‐modified CAR‐T cells exhibit T_RM_ cell characteristics and possess sustained antitumor activity.	^290^, ^291^
Radiotherapy	Rectal cancer	Radiotherapy + PD‐1/CTLA‐4	The combination of radiotherapy with CTLA‐4 and PD‐1 blockade increases T_RM_ cells within the TME.	^257^
	NSCLC	Radiotherapy + α‐PD1 + α‐MerTK mAbs	The triple therapeutic approach (Radiotherapy + α‐PD1 + α‐MerTK mAbs) enhances the presence of CD8^+^ T_RM_ cells within the microenvironment of nonlocal tumors.	^292^
Adoptive immunotherapy	Melanoma	Overexpression FOXP3 in mature CD8 T cells	FOXP3‐overexpressing CD8^+^ T cells exhibited features of T_RM_ cells and effector T cells.	^293^
Clinical trials	Vitiligo	‐	Evaluating presence and diversity of T_RM_ cells in early‐ and late‐stage vitiligo.	NCT05223738
		NB‐UVB phototherapy	Investigating how T_RM_ cell levels and phototherapy affect vitiligo lesions.	NCT05506995
	Melasma	‐	Analyzing T_RM_ cell expression in melasma lesions and its correlation with disease recurrence.	NCT05698342
	Healthy	Recombinant zoster (RZV) vaccine (Shingrix)	Assessing the impact of RZV vaccination on VZV‐specific T_RM_ cells and circulating T cells.	NCT04403139
	Healthy	Development of a methodology	Developing methods to analyze nasal T_RM_ cells and peripheral memory cells, comparing sampling devices.	NCT06469359
	Asthma	Bronchoscopy and airway brushing	Studying how memory Th2‐T_RM_ cells respond to inhaled allergens and their role in asthma.	NCT03455959
	Psoriasis	Skin (for psoriasis patients) or digestive (for IBD patients) biopsy and swab testing	Validating a tool to predict psoriasis relapse and characterizing T_RM_ cell factors.	NCT04848649
		Narrow‐band ultraviolet B (NB‐UVB) + Enstilar	Investigating the effect of NB‐UVB and Enstilar on T_RM_ cell numbers and skin microenvironment.	NCT05185258
	HIV	‐	Evaluating how circulating T cells reflect adipose tissue T‐cell distribution and insulin sensitivity.	NCT04451980

Abbreviations: EAC, esophageal adenocarcinoma; LGGs, low‐grade gliomas; NCT, neoadjuvant chemotherapy; PBC, primary biliary cholangitis.

### 5.1 Neoadjuvant therapy

Neoadjuvant cancer therapy refers to systemic treatment, including neoadjuvant chemotherapy (NAC) and neoadjuvant immunotherapy (NAI), administered before a definitive surgical operation in treatment‐naïve patients. NACs for cancer include anthracyclines (epirubicin and pirarubicin), taxanes (paclitaxel), and platinum agents (carboplatin, cisplatin, and oxaliplatin). Platinum drugs are cytotoxic DNA‐damaging compounds that can cause DNA chain breaks, potentially leading to cell apoptosis.^236^ This mechanism of action makes them particularly effective in cancer cells with DNA repair defects, such as TNBC cells carrying BRCA mutations.^237^ For patients with enriched CD8^+^CD103^+^ T_RM_ cells, platinum‐containing neoadjuvant chemotherapy agents can significantly improve the pathologic complete response (pCR) rate.^111^, ^238^, ^239^ In the context of resectable NSCLC, treatment with NAC was associated with increased numbers of CD8^+^CD103^+^ and CD4^+^CD103^+^PD‐1^+^TIM3^−^ T_RM_ cells in the TME and promoted antitumor immunity.^218^ After NAC treatment in ESCC patients, many patients exhibit increased CD103 expression within tumors, and T_RM_ cells in chemotherapy‐experienced patients exhibit improved survival compared with those in chemotherapy‐naïve patients.^119^

Immune checkpoint inhibitor (ICI) therapy has demonstrated marked clinical efficacy. Several monoclonal antibody drugs targeting CTLA‐4 and PD‐1/PD‐L1 have been developed and approved for an increasing number of indications.^240^ NAIs using ICIs have achieved clinical success in multiple tumor types, which are closely related to intratumoral T_RM_ cells.^241^ In a clinical study, preventive anti‐PD‐1 antibody treatment increased CD8^+^ T_RM_ cell infiltration into the immune microenvironment for an extended period and provided long‐term benefits to patients with ESCC.^135^, ^242^ Treatment with anti‐PD‐1 and anti‐CTLA‐4 therapy led to the expansion of intratumoral CD69^+^CD103^+^CD8^+^ T_RM_ cells, significantly increasing their cytotoxic capacity in TNBC patients and providing local immune protection against tumor rechallenge.^243^ In a preclinical murine model, compared with adoptive T_CM_ cell therapy alone, the combination of adoptive T_CM_ cells and anti‐PD‐1 antibody administration delayed the development of intradermal B16‐OVA tumors and subcutaneously injected MC38‐OVA tumors.^119^ These results showed that anti‐PD‐1 treatment expands populations of intratumoral T_RM_ cells. Similar results have also been reported for metastatic melanoma, MIBC, HNSC, NSCLC, lung adenocarcinoma, cervical cancer, gastrointestinal cancers, and nonmetastatic clear cell renal cell carcinoma.^138^, ^230^, ^244^, ^245^, ^246^, ^247^, ^248^, ^249^, ^250^ Interestingly, Rainey MA noted that NAIs could facilitate the exit of T_RM_ cells from tumors into the circulation, thereby promoting systemic tumor immunity via TGF‐β signaling.^251^

Unfortunately, ICI therapy can effectively inhibit tumor progression only in “hot” tumors (those with high immune cell infiltration). A lack of infiltrating T cells in the TME limits the antitumor effect of ICIs.^252^ “Cold” tumors (those lacking immune cells) need to be addressed with other therapies to increase immune cell infiltration in the TME, thereby transforming them into “hot” tumors and improving sensitivity to ICIs.^253^ Combining chemotherapy, radiotherapy, or chemoradiotherapy (CRT) with ICIs is a promising therapeutic strategy and is currently being used and evaluated in the clinic.^254^, ^255^, ^256^, ^257^ In patients with early‐stage TNBC, the presence of T_RM_ cells in the breast tissue is associated with improved long‐term survival following treatment with chemotherapy combined with anti‐PD‐1 therapy.^243^ CRT‐exposed TMEs are highly enriched in CD103^+^CD8^+^ T_RM_ cells in both patients with colorectal cancer and CT26 tumor‐bearing mice.^257^

### 5.2 Radiotherapy

High infiltration of CD103^+^ T_RM_ cells was strongly associated with improved prognosis in cervical tumor patients receiving radiotherapy. In a preclinical mouse model, combining HPV E6/E7‐targeted therapeutic vaccination with radiotherapy increased the number of intratumoral CD103^+^CD8^+^ T_RM_ cells.^113^ The most recent research revealed that sublethal thorax‐targeted radiation led to a rapid and sustained decline in the number of IAV‐specific lung T_RM_ cells in mice, which was associated with decreased heterosubtypic immunity. Importantly, boosting with an mRNA vaccine regenerated lung T_RM_ cells.^258^

Emerging evidence suggests that T_RM_ cells exhibit greater resistance to radiation than their circulating counterparts do. Longitudinal in vivo imaging and functional analyses have shown that a significant proportion of intratumoral T cells, including T_RM_ cells, survive clinically relevant doses of radiation. These surviving T cells display increased motility and increased production of IFN‐γ, thereby contributing to effective tumor control without the need for newly infiltrating T cells.^259^, ^260^ The interaction between T_RM_ cells and radiotherapy involves complex molecular mechanisms, including the firm adhesion of CD103 to E‐cadherin, which triggers phosphorylation cascades involving the expression of Pyk2 protein tyrosine kinase and paxillin adaptor protein. This outside‐in signaling pathway promotes the migratory behavior and effector functions of CD8^+^ T_RM_ cells, facilitating their survival and functionality postirradiation.^261^

The radioresistance of T_RM_ cells has significant implications for the design and optimization of radioimmunotherapy trials. Since T_RM_ cells are relatively resilient to radiation, local irradiation may not inherently suppress the immune response within the tumor. Instead, irradiating multiple tumors can potentially enhance systemic effects, leveraging the immunostimulatory effects of radiotherapy to amplify antitumor immunity. Additionally, transcriptomic analyses suggest that TGF‐β is a key regulator of the reprogramming of T cells within the TME, contributing to the radioresistance of T_RM_ cells.^259^ This finding opens avenues for targeting TGF‐β signaling pathways to further modulate T_RM_ cell responses and improve therapeutic outcomes.

Moreover, radiotherapy can modulate the immune microenvironment by releasing protein DAMPs from dying tumor cells, which can stimulate the sequential recruitment of neutrophils and monocytes, facilitating antitumor immune priming.^262^ Local irradiation is not inherently immunosuppressive; rather, irradiating multiple tumors might optimize the systemic effects of radiotherapy, suggesting implications for the design of radioimmunotherapy trials.

### 5.3 Vaccines

T_RM_ cells play a critical role in vaccine‐induced protective immunity and have attracted great attention in vaccine development for infection prevention and tumor immunotherapy. The relationship between vaccines and T_RM_ cells has been extensively studied in small animal and nonhuman primate models.^263^ The route of vaccine immunization has been shown to play a crucial role in the formation of T_RM_ cells. Existing data indicate that intramuscular immunization results in weaker T_RM_ cell responses in mice than mucosal or epidermal immunization does, as muscle tissue contains relatively few DCs.^264^ Accordingly, we discuss mucosal and epidermal immunization vaccines, which can generate a broad and robust T‐cell response.

A mucosal vaccine is a promising strategy for increasing the ability of mucosal T_RM_ cells to respond effectively to infections. Zens KD reported that intranasal administration of live attenuated influenza virus led to the development of CD4^+^ and CD8^+^ T_RM_ cells in the lungs, whereas systemic immunization did not achieve this in mice.^265^ Importantly, the coexpression of IL‐1β significantly increased the mucosal immunogenicity of adenoviral vector influenza vaccines.^266^ In addition, antibody‐targeted vaccination strategies can deliver antigens specifically to respiratory DCs, leading to the formation of lung CD8^+^ T_RM_ cells expressing high concentrations of GZMB and IFN‐γ, which provide strong protection against lethal influenza infections.^267^ Intranasal immunization, but not intramuscular immunization, with a chimpanzee adenoviral vector vaccine targeting the SARS‐CoV‐2 spike protein induced CD103^+^CD69^+^ T_RM_ cells activity in the lungs, effectively preventing both upper and lower respiratory tract infections caused by SARS‐CoV‐2.^268^ Intranasal vaccination with the mucosal vector B subunit of Shiga toxin generated local T_RM_ cells and suppressed HNSC growth in a mouse model.^269^ Administration of the *H. pylori* vaccine can induce the generation of antigen‐specific EGFP^+^CD4^+^ T_RM_ cells (CD69^+^CD103^−^) in the gastric subserous layer, which can reduce *H. pylori* colonization. These T_RM_ cells proliferate and differentiate in situ, enhancing local immunity during the recall response.^166^ In summary, these data suggest that mucosal vaccination against pathogens can elicit pathogen‐specific T_RM_ cells and prevent or eliminate infection at the site of entry.

Evidence has shown that epidermal disruption is the best method for eliciting a T‐cell response (including T_RM_ cells and T_CM_ cells) to vaccinia virus (VACV), which contributes to the success of the smallpox vaccine.^264^, ^270^ The modified vaccinia virus Ankara (MVA), a highly attenuated VACV, was recently approved as a smallpox vaccine. When administered via skin scarification, MVA immunization produced greater numbers of lung OVA‐specific CD8^+^ T_RM_ cells and protected mice against a lethal respiratory challenge with VACV_OVA_.^271^

In addition to vaccines, vaccine adjuvants can enhance the T_RM_ cell response generated by vaccines. The carbomer‐based nanoemulsion adjuvant system is a classic example that includes either the Toll‐like receptor 9 agonist CpG or the TLR4 agonist glucopyranosyl lipid A. Adjuvant spike protein‐based vaccines induce systemic or mucosal lung CD4^+^ T_RM_ cells and helped CD8^+^ T cells develop effective immunity to the South African B1.351 β‐variant, even without detectable mucosal or circulating virus‐neutralizing antibodies.^272^ Topical application of CpG oligodeoxynucleotides as adjuvants during subcutaneous immunization can enhance vaccine formulations by generating T_RM_ cells.^273^ In addition, the administration of a neoantigen peptide vaccine (NeoVAC) combined with α‐PD‐1 can elicit a strong neoantigen‐specific antitumor response and establish long‐term tumor‐specific immune memory in HCC by increasing CD8^+^ T_RM_ infiltration.^274^

While T_RM_ cells are crucial for tumor therapy and defense against local reinfections, they are undesirable in autoimmune diseases such as vitiligo and psoriasis, in which they contribute to pathology. Therefore, strategies to prevent the formation of new T_RM_ cells and reduce the survival of established T_RM_ cells could be beneficial in managing autoimmune diseases. Short‐term treatment with anti‐CD122 (an IL‐15 signaling inhibitor) suppresses the production of IFN‐γ by T_RM_ cells, whereas long‐term treatment eliminates T_RM_ cells from skin lesions in patients with vitiligo.^275^

### 5.4 Adoptive immunotherapy

Adoptive T‐cell therapy (ACT) is the direct infusion of cancer neoantigen‐experienced T_RM_ cells into patients suffering from cancer. In a mouse model of ACT for melanoma, CD8^+^ TILs lacking Runx3 (key regulatory factors of T_RM_ cells) expression did not accumulate within tumors, leading to increased tumor growth and heightened mortality rates. Conversely, Runx3 overexpression increased the number of T_RM_ cells, slowed tumor growth, and extended survival.^35^

A more recent branch of adoptive cell transfer is chimeric antigen receptor (CAR)‐T‐cell therapy. T_RM_ cells from patients are collected and designed to recognize and eliminate target cells by expressing a specific antigen on the cell surface.^276^ Virus‐specific CD8^+^ T_RM_ cells are present in some tumors, and these nontumor‐responsive T cells have been shown to recognize viruses.^277^ Local injection of viral peptides can reactivate these intratumoral virus‐specific CD8^+^ T cells and activate the immunosuppressive microenvironment, resulting in limited tumor growth.^123^ This finding suggests that activating tumor‐residing T_RM_ cells can contribute to tumor clearance.^278^ To date, CAR‐T‐cell therapy has been used to treat hematological malignancies but not solid tumors because it has difficulty reaching and infiltrating tumor sites.^279^ Hopefully, CAR‐T‐cell therapy can be used to increase and maintain the number of functional T_RM_ cells in solid tumors by inducing a T_RM_ cells phenotype with TGF‐β and IL‐15.^280^ In the treatment of other diseases, PD‐1‐targeting CAR‐T cells selectively deplete liver CD8^+^ T_RM_ cells and alleviate autoimmune cholangitis in an autoimmune cholangitis model.^184^ Thus, ACT and CAR‐T‐cell therapy using T_RM_ cells seem to be promising strategies for cancer therapy.

In addition to the aforementioned strategies, numerous clinical trials have been conducted in recent years to explore therapeutic approaches based on T_RM_ cells. These trials encompass a variety of treatments, including different drug combinations, immune checkpoint inhibitors, radiotherapy, and vaccines. To help readers better understand these potential therapeutic strategies and their clinical applications, we have summarized the current research progress. The key clinical trials and research findings are presented in Table 3.

## 6 CHALLENGES AND FUTURE DIRECTIONS

Research on T_RM_ cells faces several challenges and presents multiple future directions that need to be addressed. Technically, the heterogeneity of T_RM_ cells, characterized by varying surface markers and transcriptomic profiles, complicates their precise identification and classification. The nonspecificity of markers can lead to sample contamination and interpretive ambiguities. Additionally, the dynamic behavior of T_RM_ cells, including their potential migration and reentry into the bloodstream, challenges the traditional view of these cells as long‐term tissue residents. Effective targeting and modulation of T_RM_ cells for therapeutic purposes is difficult because of their developmental plasticity and varying functional states. To overcome these hurdles, future research must employ advanced experimental designs and analytical tools to deepen our understanding of T_RM_ cell biology.

Clinically, T_RM_ cells hold significant promise, especially in cancer immunotherapy. Their role in sustaining antitumor responses and their positive correlation with improved patient outcomes highlight their potential as both prognostic indicators and therapeutic targets. The ability of T_RM_ cells to operate independently of circulating T cells and respond to immune checkpoint inhibitors suggests that they could serve as predictive biomarkers for treatment efficacy. However, challenges remain in understanding T_RM_ cell differentiation, maintaining their functionality, and overcoming their potential exhaustion within tumors. Technical limitations in accurately quantifying and characterizing T_RM_ cells further complicate their study, necessitating the development of better markers and techniques.

Future research should focus on leveraging T_RM_ cells for vaccine development, particularly for infectious diseases. Innovative strategies, such as mRNA vaccines and mucosal vaccination, have shown promise in generating T_RM_ cells and enhancing immune responses. Researchers should aim to refine these approaches and explore their integration with other treatments to improve their efficacy. Additionally, understanding T_RM_ cell heterogeneity across different tissues, such as the skin, lungs, and intestines, is crucial for developing targeted vaccines and therapies. Harnessing this knowledge could lead to more effective interventions and novel therapeutic strategies, improving the functionality of T_RM_ cells and expanding their clinical applications.

## 7 CONCLUSION AND OUTLOOK

T_RM_ cells have emerged as key players in the immune system's ability to respond rapidly to recurrent infections and tumors in peripheral tissues. In this review, we comprehensively summarize the latest advances in our understanding of T_RM_ cells in various diseases and their therapeutic implications. Despite significant progress, several challenges and open questions remain. The precise mechanisms underlying the generation, maintenance, and trafficking of T_RM_ cells in different tissues and disease contexts are still poorly understood. Additionally, the heterogeneity of T_RM_ cells populations, both within and across tissues, adds complexity to their study and application. Furthermore, the influence of the TME on T_RM_ cellss’ function and survival presents significant hurdles to their successful utilization in cancer immunotherapy. The metabolic adaptability, exhaustion, and senescence of T_RM_ cells in the context of chronic inflammation or malignancy need to be further elucidated. With ongoing research efforts, we anticipate that T_RM_ cells will play an increasingly important role in the future of immunotherapy, ultimately leading to improved patient outcomes and the eradication of some of the most challenging human diseases.

## AUTHOR CONTRIBUTIONS

Daoyuan Xie, Guofeng Xu, and Guanting Lu drafted the paper and prepared figures. Qiuyan Guo and Gang Mai reviewed and edited the manuscript. All authors have read and approved the final manuscript.

## CONFLICT OF INTEREST STATEMENT

The authors declare no conflict of interest.

## ETHICS STATEMENT

Not applicable.

## Data Availability

Not applicable.
